# Attributed causes of excess mortality during the COVID-19 pandemic in a south Indian city

**DOI:** 10.1038/s41467-023-39322-7

**Published:** 2023-06-15

**Authors:** Joseph A. Lewnard, Chandra Mohan B, Gagandeep Kang, Ramanan Laxminarayan

**Affiliations:** 1 Division of Epidemiology, School of Public Health, University of California, Berkeley, Berkeley, CA USA; 2 Division of Infectious Diseases & Vaccinology, School of Public Health, University of California, Berkeley, Berkeley, CA USA; 3 Center for Computational Biology, College of Engineering, University of California, Berkeley, Berkeley, CA USA; 4 Indian Administrative Service, Chennai, India; 5 Christian Medical College, Vellore, India; 6 One Health Trust, Bangalore, India; 7 Princeton University, Princeton, NJ USA

**Keywords:** Epidemiology, Viral infection, SARS-CoV-2

## Abstract

Globally, excess deaths during 2020–21 outnumbered documented COVID-19 deaths by 9.5 million, primarily driven by deaths in low- and middle-income countries (LMICs) with limited vital surveillance. Here we unravel the contributions of probable COVID-19 deaths from other changes in mortality related to pandemic control measures using medically-certified death registrations from Madurai, India—an urban center with well-functioning vital surveillance. Between March, 2020 and July, 2021, all-cause deaths in Madurai exceeded expected levels by 30% (95% confidence interval: 27–33%). Although driven by deaths attributed to cardiovascular or cerebrovascular conditions, diabetes, senility, and other uncategorized causes, increases in these attributions were restricted to medically-unsupervised deaths, and aligned with surges in confirmed or attributed COVID-19 mortality, likely reflecting mortality among unconfirmed COVID-19 cases. Implementation of lockdown measures was associated with a 7% (0–13%) reduction in all-cause mortality, driven by reductions in deaths attributed to injuries, infectious diseases and maternal conditions, and cirrhosis and other liver conditions, respectively, but offset by a doubling in cancer deaths. Our findings help to account for gaps between documented COVID-19 mortality and excess all-cause mortality during the pandemic in an LMIC setting.

## Abstract (web summary)

Millions of excess deaths are estimated to have occurred in India during the COVID-19 pandemic, but their causes are not well documented at the national level. In this study, the authors use death registration records to describe the extent and causes of excess deaths in the large urban municipality of Madurai.

## Introduction

A reported 5.4 million people died of COVID-19 in 2020 and 2021 globally. However, this number is believed to be vastly lower than true mortality attributable to SARS-CoV-2. Excess mortality—the difference between the total number of deaths that have occurred and the number of deaths that would have been expected in the absence of the pandemic—has been used extensively to quantify the direct and indirect impacts of the pandemic^1^. According to estimates by the World Health Organization, the number of deaths occurring globally in 2020–21 exceeded pre-pandemic expectations by 14.9 million, with 86% of this burden occurring in low- and middle-income countries (LMICs) that reported smaller proportions of all confirmed COVID-19 cases and deaths^2^. In India alone, roughly 3.2–6.5 million excess deaths are estimated to have occurred through the first two waves of the COVID-19 pandemic^3,4^, a figure unmatched by any other demographic event in the country’s history since Independence in 1947^5–8^.

While understanding factors contributing to this substantial loss of life remains crucial to ongoing efforts aimed at documenting the burden of COVID-19 globally^9–12^, reliable primary data on cause-specific mortality in India and other LMIC settings remain lacking. Lessons from the COVID-19 pandemic may inform assessments of policy decisions around the implementation and relaxation of non-pharmaceutical interventions as well as both global and within-country vaccine distribution^13^. An early country-wide lockdown was effective in slowing SARS-CoV-2 transmission and blunting the first wave of COVID-19 cases;^14^ however, concerns arose that such measures—when implemented in LMIC settings including India—would lead to disproportionate harm through secondary effects on healthcare provision, food security, accidents, and interpersonal violence^15–21^. Excess death estimates encompass deaths directly caused by COVID-19 and deaths indirectly resulting from overburdening of healthcare systems with COVID-19 cases. In addition, these estimates include both increases and decreases in deaths due to other causes which may have been altered by implementation of non-pharmaceutical interventions. Whereas studies of cause-specific mortality are thus needed to better distinguish the role of COVID-19 and other factors in excess pandemic-associated mortality^22,23^, a lack of a lack of functioning vital registration systems has precluded nationwide assessments of mortality due to COVID-19 and other causes within India and most other LMIC settings^24^.

While country-wide mortality and cause-of-death surveillance are infeasible in India, subnational settings with well-functioning vital surveillance systems provide a valuable opportunity to probe changes in the frequency and causes of death during the COVID-19 pandemic. The southern state of Tamil Nadu ranks highly among Indian states in its per-capita public health investment and medical workforce, and is well regarded for the effectiveness of its primary healthcare delivery system^25^. It is also one of few Indian states with a well-performing Civil Registration System, estimated to capture 100% of deaths, based on concordance with mortality estimates from India’s parallel sample-based mortality surveillance system^26^. Madurai, a major city and administrative district within Tamil Nadu, was the site of an active SARS-CoV-2 surveillance program during the early COVID-19 pandemic which included expanded case-detection efforts through door-to-door polymerase chain reaction (PCR) testing, serological testing, and syndromic surveillance. Previous analyses of data collected through these efforts have estimated that only one in nine expected COVID-19 deaths during the first wave was captured through case-based surveillance^27^, consistent with experience in other regions of India^3,28,29^. To understand this differential between observed and expected mortality, as well as changes in mortality attributed to different causes, we analyzed Civil Registration System (CRS) records, including causes of death recorded by registered medical providers, among all decedents in Madurai over the period encompassing the initial country-wide lockdown and first two COVID-19 waves from March 2020 to July 2021.

## Results

### Continuity of mortality registration during lockdown

Because implementation of lockdown measures could impede deaths registration^30^, we first aimed to validate the tabulation of new deaths occurring during this transition period in early 2020. Whereas non-pharmaceutical interventions may have impacted cause-specific mortality beginning from March 24, 2020, changes in recorded mortality between 1 and 23 March 2020—before lockdown measures were implemented or SARS-CoV-2 transmission became widely established—would be expected to signify changes in CRS data accuracy based on reporting timelines for deaths in Madurai (see Methods). Comparing observed mortality from the “control” period of 23 March 2020 to expectations based on 2018–19 observations, the incidence rate ratio (IRR) of deaths was 1.06 (95% confidence interval: 0.94–1.20) overall, 1.11 (0.95–1.31) for males, and 1.00 (0.83–1.21) for females (Table S1). No statistically significant change was apparent for deaths occurring in healthcare facilities (IRR = 1.05 [0.84–1.31]) or in the community (IRR = 1.07 [0.93–1.24]). While this analysis does not exclude the possibility that changes in accuracy may have occurred during the later stages of the pandemic, the time immediately surrounding India’s initial country-wide lockdown was expected to be associated with the most acute interruptions in ordinary CRS procedures. Previous studies have also reported that excess mortality estimates from CRS data closely resemble findings from other (e.g., survey-based) sources in Tamil Nadu^3,31^, supporting the use of these data to analyze pandemic-associated changes in mortality within Madurai.

### Changes in all-cause mortality during the pandemic

**Fig. 1:**
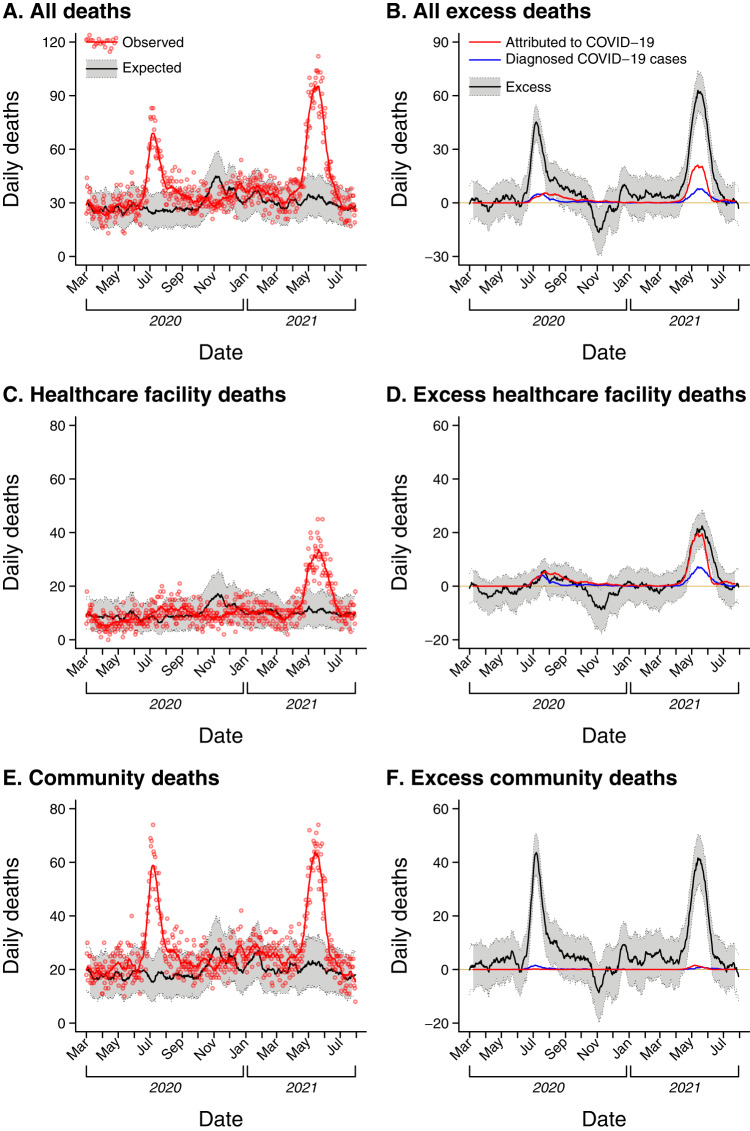
Observed and expected deaths. We plot observed and expected deaths as well as total excess deaths estimated to have occurred during the analysis period of March 2020 to July 2021, including for all settings (**A**, **B**), deaths in healthcare facilities (**C**, **D**), and deaths in community settings (**E**, **F**). Panels illustrating observed and expected deaths (**A**, **C**, **E**) present observed deaths as red points with accompanying red lines indicating 14-day moving average values. Expected deaths (sampled via Poisson distributions fitted with 2-week moving-average mortality rates from 2018–19, accounting for changes in population size [Table S19]) are presented as black lines (median estimates) along with 95% uncertainty intervals (gray shading). Panels illustrating total excess deaths (difference of observed deaths minus expected deaths; **B**, **D**, **F**) present 14-day moving average values as black lines (median estimates) along with 95% uncertainty intervals (gray shading). Accompanying red lines illustrate 2-week moving averages of total deaths attributed to COVID-19 in the medically certified cause of death data; blue lines indicate 2-week moving averages of deaths among individuals with confirmed SARS-CoV-2 infection occurring within <30 days of the positive test date. We plot corresponding age- and sex-stratified comparisons of observed and expected deaths in Fig. S1.

Projecting mortality levels from 2018–19, we expected 15,377 (14,995–15,768) deaths to occur between 1 March 2020 and 31 July 2021 (Table 1). In total, 20,004 deaths were recorded, representing a 30% (27–33%) increase over baseline expectations. Increases occurred in both medically-supervised deaths within healthcare facilities (IRR = 1.14 [1.09–1.19]) and unsupervised deaths in the community (IRR = 1.38 [1.34–1.42]; Table S2; Fig. 1). Sex-stratified analyses yielded similar findings, with deaths increasing 31% (27–36%) among males and 29% (24–34%) among females (Table S3; Table S4). These patterns varied across ages, with the greatest increases in mortality apparent among individuals aged 70–79 years and ≥80 years (44% [37–51%] and 37% [29–45%] increases, respectively), consistent with susceptibility to severe outcomes of SARS-CoV-2 infection within these age groups (Fig. S1). In contrast, fewer deaths were recorded among younger males than expected (37% [11–55%], 38% [10–57%], and 18% [–2–34%] reductions in mortality at ages 0–9 years, 10–19 years, and 20–29 years, respectively). As these changes were not apparent during the control period (Table S1), and were not reflected among females of the same ages (Table S2), interruptions in CRS function are unlikely to explain the observed reduction in mortality among boys and young adult men.

**Table 1:** Excess mortality, relative to continuation of 2018–19 pattern–both sexes

Period	Age group	Predicted (95% UI)	Observed	Excess deaths (95% UI)	Excess mortality ratio (95% UI)
Total period (1 March 2020–31 July 2021)	0–9 years	236 (182, 303)	162	−74 (−141, −20)	0.69 (0.54, 0.89)
	10–19 years	176 (133, 233)	134	−42 (−99, 1)	0.76 (0.58, 1.01)
	20–29 years	412 (345, 491)	356	−56 (−135, 11)	0.86 (0.73, 1.03)
	30–39 years	661 (580, 752)	691	30 (−61, 111)	1.05 (0.92, 1.19)
	40–49 years	1452 (1333, 1582)	1642	190 (60, 309)	1.13 (1.04, 1.23)
	50–59 years	2557 (2400, 2723)	3172	615 (449, 772)	1.24 (1.17, 1.32)
	60–69 years	3675 (3489, 3871)	4692	1017 (821, 1203)	1.28 (1.21, 1.34)
	70–79 years	3732 (3550, 3922)	5365	1633 (1443, 1815)	1.44 (1.37, 1.51)
	≥80 years	2764 (2606, 2931)	3790	1026 (859, 1184)	1.37 (1.29, 1.45)
	**All ages**	**15377 (14995, 15768)**	**20004**	**4627 (4236, 5009)**	**1.30 (1.27, 1.33)**
Early lockdown (24 March–31 May 2020)	0–9 years	38 (19, 74)	19	−19 (−55, 0)	0.49 (0.26, 1.01)
	10–19 years	26 (11, 55)	14	−12 (−41, 3)	0.55 (0.25, 1.28)
	20–29 years	44 (26, 74)	33	−11 (−41, 7)	0.75 (0.45, 1.29)
	30–39 years	97 (66, 140)	65	−32 (−75, −1)	0.67 (0.47, 0.98)
	40–49 years	190 (148, 243)	150	−40 (−93, 2)	0.79 (0.62, 1.02)
	50–59 years	333 (278, 397)	297	−36 (−100, 19)	0.89 (0.75, 1.07)
	60–69 years	473 (407, 549)	423	−50 (−126, 16)	0.89 (0.77, 1.04)
	70–79 years	479 (414, 553)	460	−19 (−93, 46)	0.96 (0.83, 1.11)
	≥80 years	363 (310, 424)	407	44 (−17, 97)	1.12 (0.96, 1.31)
	**All ages**	**2009 (1871, 2158)**	**1868**	**−141 (−290, −3)**	**0.93 (0.87, 1.00)**
Wave 1 (1 June–30 September 2020)	0–9 years	49 (28, 82)	33	−16 (−49, 5)	0.67 (0.40, 1.16)
	10–19 years	42 (22, 77)	22	−20 (−55, 0)	0.52 (0.28, 1.02)
	20–29 years	93 (66, 129)	85	−8 (−44, 19)	0.91 (0.66, 1.28)
	30–39 years	147 (114, 187)	162	15 (−25, 48)	1.11 (0.87, 1.42)
	40–49 years	331 (281, 390)	372	41 (−18, 91)	1.12 (0.95, 1.32)
	50–59 years	568 (504, 639)	723	155 (84, 219)	1.27 (1.13, 1.43)
	60–69 years	791 (719, 869)	1170	379 (301, 451)	1.48 (1.35, 1.63)
	70–79 years	789 (721, 862)	1386	597 (524, 665)	1.76 (1.61, 1.92)
	≥80 years	564 (507, 627)	995	431 (368, 488)	1.76 (1.59, 1.96)
	**All ages**	**3315 (3165, 3470)**	**4948**	**1633 (1478, 1783)**	**1.49 (1.43, 1.56)**
Wave 2 (16 March–15 July 2021)	0–9 years	60 (35, 100)	33	−27 (−67, −2)	0.55 (0.33, 0.94)
	10–19 years	40 (23, 67)	32	−8 (−35, 9)	0.80 (0.48, 1.41)
	20–29 years	90 (65, 124)	91	1 (−33, 26)	1.01 (0.73, 1.40)
	30–39 years	168 (133, 209)	198	30 (−11, 65)	1.18 (0.95, 1.48)
	40–49 years	329 (285, 378)	538	209 (160, 253)	1.64 (1.42, 1.89)
	50–59 years	572 (515, 634)	1048	476 (414, 533)	1.83 (1.65, 2.03)
	60–69 years	808 (741, 881)	1508	700 (627, 767)	1.87 (1.71, 2.04)
	70–79 years	825 (760, 894)	1797	972 (903, 1037)	2.18 (2.01, 2.37)
	≥ 80 years	621 (561, 686)	1140	519 (454, 579)	1.84 (1.66, 2.03)
	**All ages**	**3450 (3309, 3597)**	**6385**	**2935 (2788, 3076)**	**1.85 (1.78, 1.93)**

*UI* Uncertainty interval.

Excess deaths are estimated via the difference between observed deaths during 2020–21 and expected deaths for the same periods based on observations in 2018–19, accounting for projected changes in population size. Excess mortality ratios and expected mortality in the pandemic period are computed via Poisson regression models fitted to pre-pandemic (2018–19) and pandemic period (2020–21) observations, accounting for expected changes in population sizes (Table S17) via log offset terms. Bold text indicates totals across rows.

Contrary to expectations that indirect harms associated with India’s lockdown could outweigh benefits associated with blunting SARS-CoV-2 transmission^15–20^, the period from 24 March to 31 May, 2020 saw 7% (0–3%) lower-than-expected all-cause mortality (Table 1). This observation was driven by a 33% (23–42%) reduction in healthcare facility deaths, whereas medically-unsupervised community deaths did not differ appreciably from expectations (IRR = 1.06 [0.98–1.15]; Table S2). While our analyses were underpowered for demonstrating statistically-significant changes in mortality during this period within age- and sex-specific strata, apparent reductions in mortality were greatest among boys and young adult men (80% [–3–94%], 52% [–60–82%], and 28% [–49–63%] reductions at ages 0–9 years, 10–19 years, and 20–29 years, respectively; Table S3). Overall, males experienced 14% (5–22%) fewer deaths than expected during the early lockdown, whereas among females, deaths did not differ from expectations (IRR = 1.03 [0.92–1.14]) or show clear age-associated patterns of change (Table S4).

Overall, mortality was 49% (43–56%) and 85% (78–93%) higher than expected during June–September 2020 and March–July 2021, the periods encapsulating the most acute phases of the first and second waves of COVID-19 cases (Table 1). Increases in healthcare facility deaths were modest during the first wave (11% [2–21%]) in comparison to the second wave (95% [82–110%]); this difference was less clearly pronounced for medically-unsupervised deaths (68% [60–78%] and 80% [71–90%] increases during the first and second waves, respectively; Table S2). Increases in mortality during these periods were apparent among both males and females (54% [45 –63%] and 43% [33–54%] increases during the first wave, respectively, and 87% [77–97%] and 82% (71–95%) increases, respectively, during the second wave; Table S3; Table S4). The greatest increases in mortality during the two waves occurred among adults aged ≥60 years (76–118% increases over the two waves). Among children aged 0–9 years, 45% (6–67%) fewer deaths than expected were recorded during the second wave; stratifying by sex, mortality was 58% (6–79%) lower than expected among boys aged 0–9 years, whereas increases among girls of the same ages were smaller and not statistically significant (22% [–71–62%]).

### Cause-of-death assignments in CRS data

**Fig. 2:**
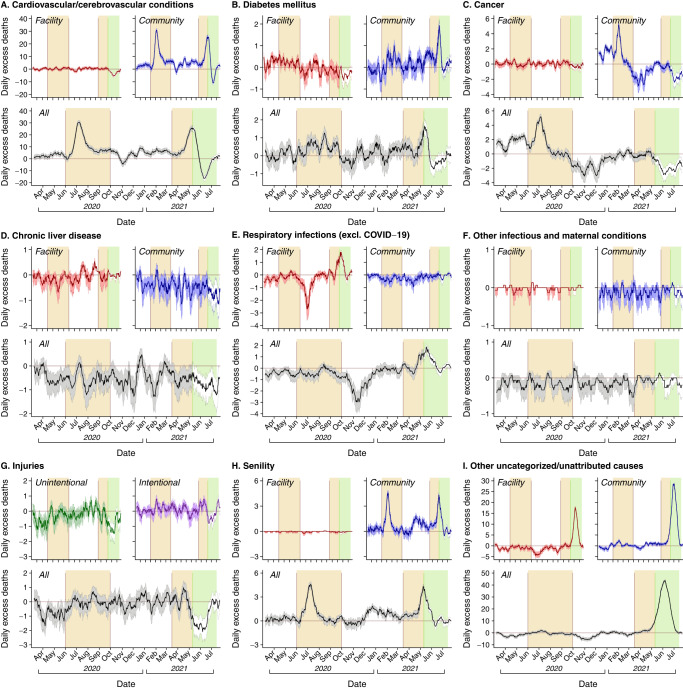
Excess deaths by attributed cause. We illustrate 2-week moving average estimates of excess deaths attributed to various causes: **A** cardiovascular and cerebrovascular conditions; **B** diabetes mellitus; **C** cancer; **D** cirrhosis and chronic liver diseases; **E** respiratory infections (excluding COVID-19); **F** other infectious and maternal conditions, besides respiratory infections; **G** injuries, **H** senility; and **I** other uncategorized or unattributed causes. Within each panel, top-left and top-right subpanels illustrate excess deaths in healthcare facilities and community (non-facility) settings, while lower subpanels illustrate all excess deaths; for injuries (**G**), we distinguish intentional and unintentional deaths in the subpanels. Lines denote median estimates; shaded areas delineate accompanying 95% uncertainty intervals, generated as draws from Poisson distributions fitted with 2-week moving-average mortality rates from 2018–19, accounting for changes in population size (Table S19). Areas with shaded backgrounds delineate the periods of the first wave (1 June to 30 September 2020) and second wave (16 March to 15 July, 2021); the green shaded area illustrates the period from 3 May 2021 onward, when uncategorized/unattributed deaths exceeded typical levels by a threefold or greater factor. To best illustrate variation in the cause-specific death attributions on the relative scale, y-axes are allowed to vary across panels due to variation in the number of deaths attributed to each cause.

We used cause-of-death data from Medically-Certified Cause of Death (MCCD) reports issued for each death in Madurai to understand how changes in all-cause mortality tracked with changes in attributions of deaths to various immediate causes. Whereas medically-certified causes of death are available for only 43.9% of deaths within Tamil Nadu (in part reflecting rural-urban differences in reporting effort)^26^, all deaths are issued medically-certified causes within Madurai; this process continued through the pandemic until late within the second wave (Fig. 2). Physicians have responsibility for reporting causes of death for patients dying under their care in healthcare facilities, and for patients who die in the community after receiving medical treatment. For deaths in the community not preceded by medical care, the registered medical provider who declares the death has the responsibility for assigning a cause of death based on their own observations or information provided by survivors and authorities who came into contact with decedents. Cause-of-death assignments in MCCD data may be imperfect; attributions to vascular diseases, cancer, respiratory diseases, and non-respiratory infections were estimated to capture 72.9%, 49.9%, 65.7%, and 53.7%, respectively, of deaths attributed to the same conditions by verbal autopsy in historical studies dating to 1995–97^32^. In contrast, a greater proportion of deaths are attributed to senility or other uncategorized causes in MCCD data^33^. Consistent with these prior observations, MCCD data from Madurai during 2018–19 attributed a lower proportion of deaths to infectious diseases, and attributed a higher proportion of deaths to uncategorized causes including senility, in comparison to verbal autopsy-based estimates from all of India^34^ (Table S5) and from other low-mortality districts^35^ (Table S6). These comparisons should be interpreted with caution due to the lack of a gold-standard reference measure for causes of death in Madurai (e.g., due to the lower burden of malaria^36^ and tuberculosis^37^ in this setting), but provide important context when interpreting cause-of-death assignments from MCCD data.

While the above factors suggest MCCD data may not reveal the same distribution of causes of death as other methods, imperfect sensitivity and specificity of cause-of-death assignments would not be expected to bias the ability of MCCD data to capture changes in cause-specific mortality over time. We therefore focused our analyses on temporal changes in death attributions. We pair our presentation of changes in cause-specific mortality with earlier findings on the reliability of cause-of-death attributions in MCCD data, and with observations from the “control” period of 1–23 March, 2020 (Table S7).

### Deaths attributed to specific causes during the pandemic period

Comparing observed data to expectations under a continuation of 2018–19 mortality levels, the period of 1 March 2020 to 31 July 2021 saw 50% (49–59%) fewer deaths attributed to infectious and maternal conditions, 18% (7–28%) fewer deaths attributed to injuries, and 21% (18–25%) more deaths attributed to noncommunicable diseases than expected (Table 2). Additionally, deaths attributed to senility and other uncategorized causes were 84% (61–112%) and 41% (33–50%) higher than expected, respectively. These patterns differed over time and across healthcare and community settings for each of the attributed causes (Fig. 2; Table S8–S12), as summarized below.

**Table 2:** Excess deaths by attributed cause—total pandemic period (1 March 2020–31 July 2021)

Cause	Predicted (95% UI)	Observed	Excess deaths (95% UI)	Excess mortality ratio (95% UI)
Infections (other than COVID-19) and maternal diseases				
Lower respiratory tract infections	354 (279, 445)	178	−176 (−267, −101)	0.50 (0.40, 0.64)
Tuberculosis	48 (28, 80)	40	−8 (−40, 12)	0.83 (0.50, 1.43)
Diarrhea and gastrointestinal infections	44 (16, 104)	11	−33 (−93, −5)	0.25 (0.11, 0.68)
Syphilis and other genitourinary diseases	28 (14, 53)	27	−1 (−26, 13)	0.97 (0.51, 1.90)
Other infectious diseases	29 (0, 196)	2	−27 (−194, 2)	0.07 (0.01, ∞)
Maternal and perinatal diseases	7 (0, 55)	2	−5 (−53, 2)	0.27 (0.04, ∞)
**Total**	**520 (427, 629)**	**260**	**−260 (−369, −167)**	**0.50 (0.41, 0.61)**
Noncommunicable diseases				
Cardiovascular/cerebrovascular conditions	8,852 (8,564, 9,149)	11,698	2,846 (2,549, 3,134)	1.32 (1.28, 1.37)
Chronic liver disease	455 (362, 569)	183	−272 (−386, −179)	0.40 (0.32, 0.51)
Cancer	855 (759, 964)	807	−48 (−157, 48)	0.94 (0.84, 1.06)
Diabetes mellitus	438 (375, 509)	537	99 (28, 162)	1.23 (1.06, 1.43)
Other noncommunicable diseases	344 (229, 506)	53	−291 (−453, −176)	0.15 (0.10, 0.23)
**Total**	**10,951 (10,621, 11,289)**	**13,278**	**2327 (1989, 2657)**	**1.21 (1.18, 1.25)**
Injuries				
Unintentional	522 (438, 620)	342	−180 (−278, −96)	0.66 (0.55, 0.78)
Intentional (suicide, homicide)	265 (216, 323)	301	36 (−22, 85)	1.14 (0.93, 1.39)
**Total**	**787 (690, 896)**	**643**	**−144 (−253, −47)**	**0.82 (0.72, 0.93)**
Other or unclassified causes				
Senility	423 (368, 485)	779	356 (294, 411)	1.84 (1.61, 2.12)
Other unclassified causes	2694 (2541, 2858)	3804	1110 (946, 1263)	1.41 (1.33, 1.50)
COVID-19	– –	1240	1240	– –

*UI* Uncertainty interval.

Excess deaths are estimated via the difference between observed deaths during 2020–21 and expected deaths for the same periods based on observations in 2018–19, accounting for projected changes in population size. Excess mortality ratios and expected mortality in the pandemic period are computed via Poisson regression models fitted to pre-pandemic (2018–19) and pandemic period (2020–21) observations, accounting for expected changes in population sizes (Table S17) via log offset terms. Bold text indicates totals across rows.

Cardiovascular and cerebrovascular conditions, which accounted for 61.5% (2,864/4,627) of all excess mortality observed, peaked in community settings during the first and second waves, but showed no overall increase in healthcare facilities throughout the pandemic (IRR = 0.95 [0.88–1.03]). Although a less common attribution, diabetes showed similar patterns. The frequency of deaths attributed to each of these conditions did not depart from expectations based on pre-pandemic mortality levels during the first weeks of India’s country-wide lockdown (IRR = 1.07 [0.97–1.17] for cardiovascular/cerebrovascular conditions and IRR = 0.92 [0.62–1.41] for diabetes), or during the control period from 1–23 March 2020 (399 deaths observed vs. 355 [303–415] expected for cardiovascular/cerebrovascular conditions; 27 deaths observed vs. 18 [10–34] expected for diabetes; Table S7), suggesting lockdown-related disruptions in routine CRS functions could not fully explain observed increases in these attributions during the first and second waves. Moreover, two-week moving average estimates of excess deaths in the community attributed to each of these conditions were positively correlated with two-week moving averages of deaths attributed to COVID-19 (Pearson’s $\rho$ = 0.64 and $\rho$ = 0.49 for cardiovascular/cerebrovascular conditions and diabetes, respectively) and deaths among individuals with confirmed SARS-CoV-2 infection ($\rho$ = 0.58 and $\rho$ = 0.59 for cardiovascular/cerebrovascular conditions and diabetes, respectively; Table 3). Furthermore, cardiovascular/cerebrovascular conditions and diabetes were commonly assigned as causes of death among individuals with confirmed SARS-CoV-2 infection (Table S13), consistent with prior evidence that these conditions are risk factors for adverse clinical outcomes of SARS-CoV-2 infection^38^, and potential sequelae of SARS-CoV-2 infection^39,40^. These observations suggest that deaths in the community attributed to cardiovascular/cerebrovascular conditions and diabetes likely included fatal, unconfirmed COVID-19 cases.

**Table 3:** Temporal association of excess mortality, by attributed cause, with deaths attributed to or associated with COVID-19

Cause	Pearson correlation coefficient, $\rho$ (95% uncertainty interval)					
	Deaths attributed to COVID-19			Deaths among confirmed COVID-19 cases		
	All deaths	Deaths in healthcare facilities	Deaths in the community	All deaths	Deaths in healthcare facilities	Deaths in the community
Infections (other than COVID-19) and maternal diseases						
Lower respiratory tract infections	0.52 (0.50, 0.54)	0.56 (0.54, 0.58)	0.09 (0.03, 0.15)	0.59 (0.57, 0.61)	0.61 (0.59, 0.63)	0.17 (0.12, 0.22)
Tuberculosis	−0.17 (−0.22, −0.12)	−0.04 (−0.09, 0.02)	−0.21 (−0.25, −0.17)	−0.10 (−0.15, −0.05)	−0.03 (−0.09, 0.02)	−0.11 (−0.16, −0.07)
Diarrhea and gastrointestinal infections	0.12 (0.08, 0.16)	−0.04 (−0.08, 0.01)	0.14 (0.09, 0.17)	0.18 (0.16, 0.20)	0.03 (0.02, 0.05)	0.18 (0.16, 0.20)
Syphilis and other genitourinary diseases	0.01 (−0.04, 0.05)	−0.12 (−0.18, −0.07)	0.08 (0.03, 0.12)	0.06 (0.01, 0.10)	−0.02 (−0.05, 0.01)	0.08 (0.03, 0.13)
Other infectious diseases	0.08 (0.03, 0.13)	−0.09 (−0.17, −0.02)	0.14 (0.10, 0.17)	0.08 (0.03, 0.12)	−0.11 (−0.20, −0.03)	0.14 (0.12, 0.16)
Maternal and perinatal diseases	0.04 (0.00, 0.08)	0.02 (−0.01, 0.05)	0.04 (−0.01, 0.09)	−0.01 (−0.07, 0.04)	0.01 (−0.02, 0.03)	−0.02 (−0.10, 0.05)
**Total**	**0.67 (0.66, 0.69)**	**−0.16 (−0.23, −0.09)**	**0.69 (0.68, 0.70)**	**0.55 (0.53, 0.56)**	**−0.19 (−0.27, −0.11)**	**0.56 (0.55, 0.58)**
Noncommunicable diseases						
Cardiovascular/cerebrovascular conditions	0.54 (0.53, 0.56)	−0.22 (−0.24, −0.19)	0.64 (0.63, 0.65)	0.52 (0.50, 0.52)	−0.09 (−0.12, −0.06)	0.58 (0.57, 0.59)
Chronic liver disease	−0.03 (−0.08, 0.02)	−0.07 (−0.11, −0.03)	0.02 (−0.03, 0.08)	−0.03 (−0.09, 0.02)	−0.03 (−0.07, 0.01)	−0.02 (−0.08, 0.04)
Cancer	0.04 (0.02, 0.05)	−0.22 (−0.27, −0.17)	0.08 (0.07, 0.10)	−0.11 (−0.13, −0.10)	−0.16 (−0.21, −0.10)	−0.09 (−0.11, −0.07)
Diabetes mellitus	0.35 (0.31, 0.39)	−0.15 (−0.20, −0.11)	0.49 (0.46, 0.52)	0.41 (0.37, 0.44)	−0.20 (−0.25, −0.16)	0.59 (0.56, 0.62)
Other noncommunicable diseases	−0.09 (−0.12, −0.06)	0.03 (−0.03, 0.08)	−0.09 (−0.13, −0.06)	−0.20 (−0.24, −0.17)	−0.02 (−0.08, 0.04)	−0.20 (−0.24, −0.17)
**Total**	**0.54 (0.53, 0.56)**	−**0.22 (**−**0.24**, −**0.19)**	**0.64 (0.63, 0.65)**	**0.52 (0.50, 0.52)**	−**0.09 (**−**0.12**, −**0.06)**	**0.58 (0.57, 0.59)**
Injuries						
Unintentional	−0.31 (−0.35, −0.26)	−0.22 (−0.27, −0.17)	−0.26 (−0.31, −0.21)	−0.23 (−0.28, −0.19)	−0.13 (−0.17, −0.08)	−0.25 (−0.30, −0.20)
Intentional (suicide, homicide)	−0.19 (−0.24, −0.14)	0.01 (−0.05, 0.07)	−0.20 (−0.24, −0.15)	−0.19 (−0.24, −0.14)	0.04 (−0.02, 0.09)	−0.20 (−0.25, −0.15)
**Total**	**−0.31 (−0.35, −0.26)**	**−0.22 (−0.27, −0.17)**	**−0.26 (−0.31, −0.21)**	**−0.23 (−0.28, −0.19)**	**−0.13 (−0.17, −0.08)**	**−0.25 (−0.30, −0.20)**
Other or unclassified causes						
Senility	0.67 (0.66, 0.69)	−0.16 (−0.23, −0.09)	0.69 (0.68, 0.70)	0.55 (0.53, 0.56)	−0.19 (−0.27, −0.11)	0.56 (0.55, 0.58)
Other unclassified causes	0.58 (0.58, 0.58)	0.40 (0.39, 0.41)	0.66 (0.65, 0.66)	0.45 (0.44, 0.45)	0.23 (0.22, 0.24)	0.55 (0.55, 0.56)

Values indicate Pearson correlation coefficients relating two-week moving averages of excess deaths for each attributed cause, stratified by setting, to two-week moving averages of variables measuring deaths attributed to COVID-19 in medically-certified cause-of-death data (left columns) and deaths occurring within 30 days after a positive test result for confirmed COVID-19 cases (right columns). Uncertainty intervals are generated via independent draws from the distribution of the cause-specific excess mortality variable, generated by sampling from Poisson distributions parameterized with 2018–19 mortality rates and accounting for expected changes in population size (Table S19). Bold text indicates totals across rows.

Deaths attributed to senility increased 147% (94–214%) and 117% (71–177%), respectively, during the first and second waves (Table S9, Table S10), with nearly all deaths receiving this attribution occurring in community settings (99.6% [776/779]; Table S11, Table S12). Moderate increases in such attributions were also noted during early phases of the lockdown (IRR = 1.49 [1.05–2.15]), whereas other uncategorized causes of death were assigned less frequently during the same period (IRR = 0.61 [0.50–0.74]; Table S8). As these changes were directionally consistent with observations during the control period from 1–23 March 2020 (28 deaths observed vs. 21 [11–38] expected for senility; 91 deaths observed vs. 108 [78–174] expected for other uncategorized causes), our findings suggest that implementation of lockdown measures contributed to increases in the use of these non-specific attributions in MCCD data. By the time of the second wave, 33.3% of all deaths observed (2127/6385) were not attributed to specific causes (IRR = 3.64 [3.35–3.96). This change was apparent for both medically-supervised deaths and those occurring in the community, potentially leading to undercounting of deaths due to other factors during acute phases of the second wave (Fig. 2). Overall, senility and uncategorized causes were indicated for 14.2% of decedents with confirmed SARS-CoV-2 infection (82/578; Table S13), and two-week moving averages of excess mortality attributed to both causes were correlated with deaths attributed to COVID-19 ($\rho$ = 0.67 and $\rho$ = 0.58 for senility and other uncategorized causes, respectively) and with deaths among individuals with confirmed SARS-CoV-2 infection ($\rho$ = 0.55 and $\rho$ = 0.45 for senility and other uncategorized causes, respectively; Table 3). Thus, attributions of deaths to senility and other uncategorized causes may have increased in association with implementation of lockdown measures, and may encompass deaths among fatal, unconfirmed COVID-19 cases.

Total deaths attributed to infectious diseases and maternal conditions were 61% (19–79%) lower than expected during the first weeks of lockdown, and 42% (11–62%) lower than expected during the first wave; by the time of the second wave, deaths attributed to infectious and maternal conditions were 29% (–4–73%) higher than expected (Table S8, Table S9, Table S10). Incidence rate ratios for deaths attributed to lower respiratory tract infections, which accounted for 68.4% of deaths in this category (178/260), were 0.25 (0.09–0.81) during the early lockdown, 0.59 (0.34–1.05) during the first wave, and 1.67 (1.21–2.34) during the second wave. Reductions were apparent for medically supervised deaths attributed to lower respiratory tract infections both during the early lockdown and during the months of October 2020 through January 2021, corresponding to the season when such deaths are typically more common (Fig. 2). However, the number of deaths attributed to infectious and maternal conditions was also lower than expected in the pre-lockdown control period (3 observed vs. 21 [0–100] expected), making it unclear whether observed patterns reflected true reductions in infectious disease burden, as reported in other settings^41–45^, or changes in death attributions associated with lockdown-related disruptions to MCCD functions.

Increases in deaths attributed to lower respiratory tract infections during the second wave were observed only in healthcare settings (Fig. 2). Moreover, time series of COVID-19–related deaths were more strongly associated with deaths attributed to lower respiratory tract infections occurring in healthcare settings in comparison to community settings ($\rho$ = 0.56 and $\rho$ = 0.09, respectively, for the association with deaths attributed to COVID-19; $\rho$ = 0.61 and $\rho$ = 0.17, respectively, for the association with deaths among individuals with confirmed SARS-CoV-2 infection; Table 3). These outcomes may reflect attributions of some COVID-19 deaths to lower respiratory tract infections generally, as reported in other settings^46^, or may indicate the contribution of secondary respiratory infections such as mucormycosis to fatal outcomes among individuals with confirmed or unconfirmed SARS-CoV-2 infection, especially in healthcare settings.

Both unintentional and intentional injuries declined during the first weeks of lockdown (IRR = 0.40 [0.24–0.67] and 0.80 [0.44–1.54], respectively), whereas injury-associated mortality during the control period from 1–23 March 2020 did not differ from expected levels (38 deaths observed vs. 35 [21–58] deaths predicted; Table 2; Table S7; Table S8). Whereas deaths attributed to intentional injuries returned to expected or higher-than-expected levels during the first and second pandemic waves (IRR = 1.28 [0.89–1.87] and 1.19 [0.81–1.76], respectively), deaths attributed to unintentional injuries remained lower than expected throughout these periods (IRR = 0.72 [0.51–1.02] and 0.41 [0.28–0.61], respectively; Table S9, Table S10). Injury-attributed deaths matched expected levels in the community (IRR = 1.01 [0.86–1.18]), with the observed decrease fully accounted for by reductions in medically-supervised deaths (IRR = 0.54 [0.43–0.69]; Table S11, Table S12). This observation suggests that individuals with injuries who would ordinarily have received healthcare may have instead died in the community, possibly reflecting reduced access to healthcare facilities due to burden associated with managing COVID-19 patients, as suggested in other settings within India^47^.

Deaths attributed to cirrhosis and other liver diseases, a majority of which are related to alcohol consumption in India^48^, likewise declined by 76% (43–88%) during the early lockdown and remained at lower-than-expected levels throughout the first and second waves (IRR = 0.32 [0.21–0.53] and 0.41 [0.27–0.65], respectively; Fig. 2; Table S8; Table S9; Table S10). These changes may correspond to India’s ban on alcohol sales during the initial lockdown and subsequent periods of intensified non-pharmaceutical interventions in response to surges; prior studies have associated these measures with increases in alcohol withdrawal and related outcomes^49,50^. As reductions in deaths attributed to cirrhosis and other liver diseases were observed both in healthcare facilities (IRR = 0.69 [0.49–0.99]; Table S11) and the community (IRR = 0.30 [0.22–0.40]; Table S12), and changes were not apparent during the control period from 1–23 March 2020 (11 observed vs. 14 [5–33] predicted deaths), disruptions in MCCD procedures or in cirrhosis and liver disease diagnoses were unlikely to account for these observations.

Last, deaths attributed to cancer increased 109% (63–169%) during the first weeks of lockdown, and remained 20% (0–45%) above expected levels through the first wave (Table S8; Table S9). These observations were driven by excess cancer deaths occurring in the community during early phases of the pandemic (Fig. 2). By the time of the second wave, however, cancer deaths were 43% (23–57%) lower than expected (Table S10). This observation may reflect a “harvesting” effect^51^ resulting from rapid clinical deterioration among existing cases, as well as reductions in new cancer diagnoses due to interruptions in routine care during the pandemic. Prior studies have reported reductions in care quality for cancer patients (e.g., fewer follow-up visits, fewer chemotherapy courses administered, and fewer surgeries performed) as well as reduced cancer screening and diagnosis of new cases within India during the pandemic^52–54^.

### Associations of excess mortality with community deprivation

**Fig. 3:**
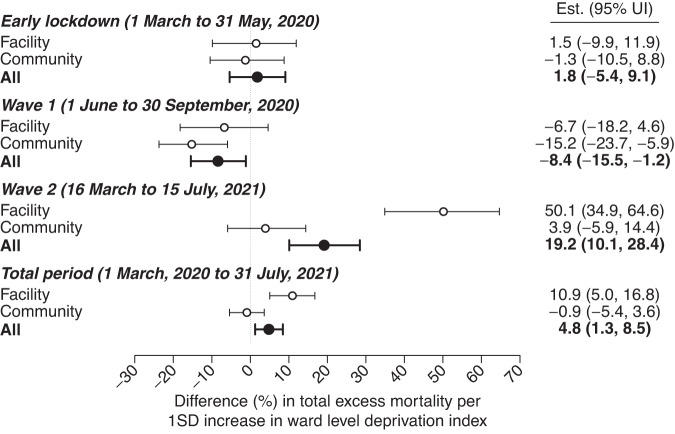
Association of excess deaths with ward-level deprivation indicators. We illustrate estimates of the association between excess deaths and a ward-level measure of community deprivation, constructed as the first principal component of 15 socioeconomic indicators measured in the 2011 Census of India (Table S14). Values correspond to the absolute difference (in percentage-point units) in excess mortality, measured relative to expected deaths, associated with an increase by one standard deviation in the principal component-based measure of community deprivation. Lines denote 95% uncertainty intervals surrounding point estimates (medians), as estimated across regression models fitted across 10,000 independent draws from the distribution of the excess mortality outcome variable.

Last, to understand the role of socioeconomic factors in observed changes in mortality, we constructed a ward-level deprivation index using the first principal component extracted from a set of indicators measured in the most recent (2011) Census of India (Table S14). On average, each increase by one standard deviation in the ward-level deprivation measure was associated with 5% (1–9%) higher levels of overall excess all-cause mortality, and 11% (5–17%) higher levels of deaths in healthcare settings, during the pandemic period (Fig. 3). Socioeconomic disparities in excess mortality were most pronounced during the second wave, when each one-standard-deviation increase in deprivation was associated with 19% (10–28%), 50% (35–65%), and 4% (–6–14%) higher excess mortality overall, in healthcare facilities, and in the community, respectively. In contrast, wards with greater socioeconomic deprivation experienced modestly lower excess mortality during the first wave across both community and healthcare settings. Changes in mortality during the lockdown were not clearly associated with community deprivation measures. These patterns held in analyses relating all-cause mortality to most of the specific census indicators used to generate the community deprivation index (Fig. S2).

Wards with greater degrees of deprivation experienced greater excess mortality attributed to infectious diseases and maternal conditions, and greater excess mortality attributable to noncommunicable diseases, throughout the total pandemic period and during the second wave, in particular (Table S15). In contrast, community deprivation was not clearly associated with excess mortality attributed to other causes, although these analyses encountered limited statistical power in comparison to those addressing excess all-cause mortality. Measures aiming to capture the completeness of COVID-19 death reporting, including the ratio of deaths among individuals with documented SARS-CoV-2 infection to all-cause excess deaths, and the ratio of deaths attributed to COVID-19 to all-cause excess deaths, were not clearly associated with community deprivation indicators (Table S16).

## Discussion

Globally, excess mortality during the first two years of the COVID-19 pandemic exceeded deaths attributed to COVID-19 by nearly three-fold (14.9 vs. 5.4 million through 31 December 2021)^2^. The majority of unaccounted-for deaths are believed to have occurred in LMIC settings, where excess mortality over this period amounted to 12.7 million deaths, in contrast to 1.2 million deaths reported among confirmed COVID-19 cases. India alone accounts for 3.2–6.5 million excess deaths through 2021, representing 21–44% of the estimated total globally. Although India is a diverse country with significant variation in health systems, demographics, and completeness of civil registration records, findings from our analyses may provide insight into changes in mortality with implications extending beyond Madurai, where continuity of CRS and MCCD functions uniquely enabled our study. Discrepancies between all-cause and reported COVID-19 mortality in Madurai closely resemble findings in other settings within India, both over time and across demographic strata^3,27,55–58^.

While all-cause deaths increased 30% overall during the period from March 2020 to July 2021, excess deaths were concentrated during periods of peak SARS-CoV-2 transmission associated with the ancestral and Delta (B.1.617.2) variants. The first and second waves saw 25 and 32% of all deaths occurring during the study period, respectively, and 35 and 63% of all excess deaths estimated to have occurred, respectively; mortality occurring during the intervening periods was slightly lower than expected. Excess deaths attributed to infectious and maternal conditions—most prominently including lower respiratory tract infections—as well as cardiovascular/cerebrovascular conditions, diabetes, senility, and other uncategorized causes, exhibited strong temporal associations with surges in deaths attributed to COVID-19 or occurring among individuals with confirmed SARS-CoV-2 infection. Thus, these findings may reveal attributed causes of death among fatal COVID-19 cases, a majority of which likely went undiagnosed.

In contrast, deaths attributed to several other causes, including unintentional injuries and cirrhosis and related liver conditions, exhibited sustained reductions throughout the pandemic which were not observed during a “control” period from 1–23 March 2020, when lockdown-associated disruptions may have been expected to impact reporting. Non-pharmaceutical interventions may have played a role in reducing individuals’ risk of death due to these causes, consistent with findings in several high-income settings^59^. Implementation of lockdown measures accompanied expanded social welfare programs targeting older adults and other vulnerable populations in Madurai and other regions of India; these included increasing food rations for individuals covered by the National Food Security Act and door-to-door mobilization of social and community healthcare workforces to deliver essential supplies during the lockdown as well as screen individuals for symptoms and infection^27^. These efforts may have helped to mitigate vulnerable individuals’ risk of death due to causes unrelated to COVID-19.

While the 30% increase in deaths in Madurai due to all causes is consistent with observations in other settings across India^3,30^, this total greatly exceeds global average increases of 8% in 2020 and 18% in 2021^4^. Other settings with >25% increases in mortality during the pandemic have included Russia and LMICs within Latin America, Eastern Europe, and central Asia^1,4^. As total excess mortality encompasses both COVID-19 deaths as well as increases and decreases in deaths due to causes unrelated to COVID-19, it is important to note that excess mortality ratios do not directly measure the burden of deaths attributable to COVID-19 in any setting.

While excess medically-supervised deaths attributed to lower respiratory infections varied in association with deaths among confirmed COVID-19 cases and deaths attributed to COVID-19, excess deaths attributed to cardiovascular/cerebrovascular conditions, diabetes, and senility or other uncategorized causes occurred primarily in community settings. Such attributions should be viewed with particular scrutiny, as medical practitioners issuing cause-of-death assignments for individuals who died in the community without prior medical care may have had limited access to clinical information to inform their assessment; attributions to senility and uncategorized causes pose particular risks of inaccuracy^33,60^. Deaths for which these causes were assigned could also have increased in the community as a result of avoidance of healthcare settings, or due to patients’ lack of access to healthcare facilities during periods with substantial COVID-19 caseload.

Our study has several limitations. First, cause-of-death determinations in MCCD data are expected to be imperfect^33,61^. Our findings should thus be interpreted as representing changes in assigned causes of death over time rather than characterizing the true distribution of causes of death within Madurai. Many deaths were not assigned causes at the height of the second wave, when the number of deaths occurring overwhelmed local health and vital surveillance systems. While our study benefits from continuous mortality records in 2018–19 to define baseline expectations, unstable counts for rare death attributions and in small age- or sex-specific strata may limit our ability to reliably predict expected mortality levels in 2020–21. Analyses of ward-level socioeconomic characteristics associated with excess mortality are likewise limited by the lack of updated Census of India data since 2011. As slums or makeshift settlements may be closely interspersed with higher-income communities, analyses undertaken at the level of city wards, and drawing on older data on socioeconomic characteristics of wards, may mask the full extent of socioeconomic variation in excess and cause-specific mortality associated with the COVID-19 pandemic. While the population of Madurai Corporation was estimated at 1,017,865 as of the 2011 census, a lack of reliable up-to-date estimates of population size, overall and within age- and sex-specific strata, and uncertainty about migration which may have occurred during the pandemic in association with lockdown measures, precluded direct estimation of per-capita mortality associated with excess COVID-19 deaths. Last, although it is reassuring that age- and sex-specific observations of all-cause mortality in Madurai broadly reflect those reported in other settings within India^3,28,57^, Madurai is only one city, and observations in this setting may not be uniformly generalizable across regions or to rural contexts. For example, whereas maternal and perinatal conditions are not a major cause of death in Madurai, poorer and rural regions of the country experience markedly higher maternal and infant mortality^62^. Notwithstanding these limitations, Madurai presents an important setting for analyses, as CRS data have limited reliability throughout much of the rest of the country. Data on medically-certified causes of deaths are even more scarcely available within other Indian states and LMICs, where understanding of the impact of the COVID-19 pandemic on cause-specific mortality remains limited.

Correctly accounting for deaths and their causes during acute emergencies carries important societal ramifications^9–12^. While mitigation of COVID-19 morbidity and mortality during the first wave was largely dependent upon non-pharmaceutical interventions, administration of the ChAdOx1 vaccine in India began 16 January 2021, prior to the Delta variant surge. Effectiveness of ChAdOx1 against Delta variant-associated severe disease has been estimated at ≥80% in multiple postlicensure studies^63,64^, including in India^65^. Thus, a majority of COVID-19 deaths during India’s second wave could have been programmatically preventable under a scenario with improved vaccine coverage. Reduced burden on healthcare systems may have also helped to prevent mortality associated with causes unrelated to COVID-19. While our study identifies that lockdowns may have been associated with increases in deaths due to cancer and other conditions for which treatment was interrupted, it is important to note that all-cause mortality declined during this period in Madurai, consistent with observations elsewhere in India^57^. This finding, together with prior evidence of the effectiveness of India’s nationwide lockdown in delaying widespread SARS-CoV-2 transmission^14^, presents a reassuring contrast to expectations in early 2020 that lockdowns could cause greater harm than public health benefit, although measures of impact besides mortality remain important to consider.

India is one of many settings where excess all-cause mortality during the pandemic vastly exceeded reported COVID-19 deaths^1^. While such gaps have received particular attention in LMICs^66–68^, where surveillance and vital registration systems may encounter particular strain, the need to reconcile COVID-19 deaths with all-cause excess mortality has also arisen in high-income countries^69–71^. Our findings support ongoing efforts to quantify mortality associated with the COVID-19 pandemic globally, to identify causes of systematic undercounting, and to evaluate the impact of COVID-19 lockdown measures on mortality associated with various causes. Changes in cause-specific mortality during acute phases of the COVID-19 pandemic should inform planning for future public health emergencies necessitating non-pharmaceutical interventions.

## Methods

### Civil registration of deaths

Data for this study were generated through routine surveillance via CRS and MCCD functions within the municipality of Madurai (Madurai Corporation), including MCCD reporting. Vital surveillance in India is mandated under the Registration of Births and Deaths Act of 1969, which provides standardized elements for reporting births and deaths. Operating procedures of the CRS across India are decentralized, with the expectation that local registration units (states and districts) develop customized strategies adapted to their unique contexts. In Tamil Nadu, a local Coordinating Committee leads vital surveillance within districts. Heads of affected households, in coordination with executive officers of lower administrative units (e.g., taluks, blocks, wards, or villages) have legal responsibility for notification of all births and deaths occurring in the community to their local registration unit. Police, community healthcare workers, operators of crematories or cemeteries, and other officials who come into contact with the deceased have a responsibility to report deaths that they are the first to observe. Under the MCCD system, attending physicians have responsibility for reporting deaths occurring in healthcare facilities under their supervision, and for assigning medically-certified causes of death to these patients. In addition, physicians who provide care to individuals who die in the community have the responsibility for assigning causes of death; causes of death for individuals who die in the community without prior care are assigned by registered medical providers at the point of declaring each death^72^. Data included unique records for each death within Madurai Corporation during the periods of interest (2018–19 and 2020–21) abstracted from standardized MCCD forms (Table S17) and CRS forms (Table S18) including, for each decedent, their age, sex, date of death, ward of residence, attributed immediate cause of death, and name of the facility where the death occurred (for deaths occurring in healthcare facilities). Recording of fatal outcomes among individuals with SARS-CoV-2 infection has been described previously for this setting^27^. Death registrations in MCCD data for confirmed COVID-19 cases who experienced fatal outcomes within ≤30 days of a positive test outcome were linked via manual record review.

### Analytic framework

We aimed to compare observed mortality during the pandemic period (2020–21) to expectations under a status-quo scenario of pre-pandemic mortality levels observed in 2018–19. Periods of interest for analysis included the total pandemic period (1 March 2020 to 31 July 2021); early lockdown period (24 March–31 May 2020); first wave (1 June–30 September 2020); and second wave (16 March–15 July 2021). For each period, we summed total deaths across the applicable date range during the pandemic (2020–21) and pre-pandemic (2018–19) periods. We also defined a “control” period from 1–23 March 2020 to assess changes in reporting potentially associated with implementation of non-pharmaceutical interventions. Whereas deaths during 1–23 March 2020 are not likely to have been attributable to COVID-19, and preceded implementation of non-pharmaceutical interventions, reporting of deaths occurring during this period could have been affected by acute lockdown-associated disruptions (for deaths between 1 January and 29 February 2020, median time to registration was 12 days, and 30.8% were registered >21 days after occurring). We, therefore, aimed to compare observed to expected mortality during this period to determine whether implementation of lockdown measures was associated with changes in deaths reporting.

### Statistical analysis

For primary analyses of excess all-cause mortality, we fit Poisson regression models to total mortality counts for each period of interest; data included period-specific total mortality during the applicable date ranges in 2018, 2019, 2020, or 2021. For each period, we defined population offsets accounting for expected changes in age- and sex-specific population sizes (log-transformed) for 2020–21 (Table S19). We used this framework to estimate the IRR of mortality comparing the pandemic period to 2018 and 2019, accounting for interannual variability during these years as well as expected changes in population size based on estimated year-on-year changes in population within age- and sex-specific population strata for Madurai District. To quantify absolute mortality expectations under a continuation of pre-pandemic mortality levels, we sampled from a Poisson distribution with a rate parameter defined as the product of the fitted model intercept (corresponding to averaged mortality rates during 2018 and 2019) and person-time at risk within the pandemic period. We subtracted these results for projected mortality from observed mortality to quantify absolute excess mortality. We used the same framework to compare observed mortality within age- and sex-specific population strata, and mortality attributed to specific causes, against expectations based on pre-pandemic observations.

To understand the potential association of various attributed causes of death with unconfirmed, fatal COVID-19 cases, we next aimed to determine whether cause-specific excess death time series were correlated with two measures of mortality related to COVID-19: (1) the number of deaths attributed to COVID-19 in MCCD data, and (2) the number of deaths occurring among individuals with confirmed SARS-CoV-2 infection. We generated 2-week moving average time series of expected mortality for all causes and for specific causes, in both healthcare and community settings, by sampling from Poisson distributions for total deaths each calendar day; these Poisson distributions were parameterized by mortality rates over the period from 7 days before to 7 days after each calendar date. We defined two-week moving averages of excess mortality by subtracting sampled draws from the distribution of expected mortality over each period 7 days before to 7 days after each period of interest from observed mortality over the same period. We then computed Pearson correlation coefficients measuring the association of the resampled time series of 2-week moving averages of excess mortality with each of the two independent variables measuring COVID-19-related mortality.

We also aimed to measure associations of excess mortality with socioeconomic characteristics of communities within Madurai. We used data from the 2011 Census of India to characterize socioeconomic attributes of wards within Madurai (*N* = 100); whereas the census is ordinarily carried out on a decennial basis, data collection for the 2021 Census of India was delayed to 2023 due to disruptions from the COVID-19 pandemic. Consistent with prior analyses^57^, extracted variables included the following: household crowding (measured as the mean number of individuals per room within a household); adult illiteracy (as a proportion among all adults); membership in scheduled castes or tribes (measured as a proportion among all adults); household condition (measured as the proportion of households classified as residing within dilapidated structures, within non-permanent structures, within structures with unfinished flooring, within structures without electric lighting, or reliant on solid cooking fuels); water and sanitation access (measured as the proportion of households lacking tapped, treated water sources, without onsite water sources, without onsite latrines, without onsite sewer connections, or reliant on open defecation); access to banking (measured as the proportion of households with bank accounts); and lack of material assets (measured as the proportion of households lacking each of the following index assets: computer, phone, bicycle, scooter/moped/motorcycle, and car/jeep van; Table S14). We generated a single index measuring community disadvantage as the first principal component of all extracted variables. The first principal component accounted for 48% of variation across all indicators and was positively associated with each measure besides household crowding at the two-sided *p* < 0.05 threshold.

We quantified associations of excess deaths with socioeconomic characteristics of communities via a regression model defining log-transformed mortality IRRs during each period (as estimated using the Poisson regression framework described above), within each ward, as the outcome, and the community deprivation index as the exposure. To propagate uncertainty in ward-level excess mortality, we repeated regression analyses across 10,000 samples from the distribution of excess mortality estimates by ward. As a secondary analysis, we also estimated associations of excess ward-level mortality with each of the deprivation indicators used to generate the principal component-based index individually, applying the same regression framework.

Finally, we aimed to assess whether community socioeconomic attributes were associated with the completeness of COVID-19 mortality reporting. We assessed reporting completeness using case-based and mortality-based surveillance, defining two independent variables as potential measures of completeness: (1) the proportion of all excess deaths occurring among reported cases within each ward, obtained by dividing ward-level deaths among confirmed COVID-19 cases by total excess deaths; (2) the proportion of all excess deaths attributed (on death certificates) to COVID-19, obtained by dividing ward-level deaths attributed to COVID-19 by total excess deaths. Each of these analyses propagated uncertainty in excess death measures according to the framework described above; parameter estimates are pooled across analyses undertaken on unique draws from the distribution of excess mortality by ward.

### Ethics and inclusion

Local authors (C.M.B., G.K., R.L.) were involved in all components of the research process including study conception in relation to local priorities, study design, and authorship of publications. Analyses of de-identified mortality data generated through routine vital surveillance functions of the Civil Registration System were considered to constitute non-human subjects research and ethical review of the project was not required by local institutions (Madurai Municipal Corporation) as a condition for data sharing, or by foreign institutions with which the authors are affiliated (University of California, Berkeley; Princeton University) as a condition for data analysis (NIH Exemption Category 4 for non-human subjects research). This research project did not involve risks to human or animal subjects or environments, or international transfer of biological materials or cultural artefacts from India. Studies undertaken in India have been cited appropriately.

### Reporting summary

Further information on research design is available in the Nature Portfolio Reporting Summary linked to this article.

## Supplementary information

Supplementary Information

Peer Review File

Reporting Summary

## Data Availability

De-identified individual-level mortality data reported in this study are available via GitHub (https://github.com/joelewnard/Madurai_Deaths)^73^. Census of India data are available publicly available from https://censusindia.gov.in/census.website/.

## Code availability

Analysis code is available from GitHub (https://github.com/joelewnard/Madurai_Deaths)^73^.
