# Supplementary material for: Attributed causes of excess mortality during the COVID-19 pandemic in a south Indian city
Source: Nat Commun. 2023 Jun 15;14:3563. doi: 10.1038/s41467-023-39322-7 (PMC10272147; doi:10.1038/s41467-023-39322-7)
Supplement: Supplementary file 1 — Supplementary Information [file 41467_2023_39322_MOESM1_ESM.pdf]

Supporting information

Attributed causes of excess mortality during the COVID-19 pandemic in a south Indian city

Joseph A. Lewnard[1,2,3,*], Chandra Mohan B.[4], Gagandeep Kang[5], Ramanan Laxminarayan[6,7]

1. Division of Epidemiology, School of Public Health, University of California, Berkeley, Berkeley, California, United States
2. Division of Infectious Diseases & Vaccinology, School of Public Health, University of California, Berkeley, Berkeley, California, United States
3. Center for Computational Biology, College of Engineering, University of California, Berkeley, Berkeley, California, United States
4. Government of Tamil Nadu, Chennai, India
5. Christian Medical College, Vellore, India
6. One Health Trust, Bangalore, India
7. Princeton University, Princeton, New Jersey, United States

Contents of this supplement

Item	Title	Page
Table S1	Excess deaths, relative to continuation of 2018-19 pattern, during control period (1 March to 23 March, 2020).	2
Table S2	Excess mortality by healthcare facility or community setting.	3
Table S3	Excess deaths, relative to continuation of 2018-19 pattern—Males.	4
Table S4	Excess deaths, relative to continuation of 2018-19 pattern—Females.	5
Table S5	Comparison of attributed causes of death during the pre-pandemic period to verbal autopsy-based estimates for all of India, 2017-2019, by age and sex.	6
Table S6	Comparison of attributed causes of death during the pre-pandemic period to verbal autopsy-based estimates, for adults aged 15-69 years in low-mortality districts, 2014.	7
Table S7	Excess deaths by attributed cause—Control period (1 March–23 March, 2020).	8
Table S8	Excess deaths by attributed cause— Early lockdown (24 March–31 May, 2020).	9
Table S9	Excess deaths by attributed cause—Wave 1 (1 June–30 September, 2020).	10
Table S10	Excess deaths by attributed cause—Wave 2 (16 March–15 July, 2021).	11
Table S11	Excess deaths by attributed cause—Deaths occurring in healthcare facilities during the total pandemic period (1 March, 2020–31 July, 2021).	12
Table S12	Excess deaths by attributed cause—Deaths occurring in the community during the total pandemic period (1 March, 2020–31 July, 2021).	13
Table S13	Reported causes of death among individuals with or without documented SARS-CoV-2 infection.	14
Table S14	Association of ward-level characteristics with deprivation index.	15
Table S15	Association of cause-specific excess deaths with ward-level deprivation index.	16
Table S16	Association of the proportion of excess deaths attributed to COVID-19 with ward-level deprivation index.	17
Table S17	Data elements of the medical certificate of cause of death for hospitalized patients (Form 4) and non-institutional deaths (Form 4A).	18
Table S18	Data elements of the death registration form.	19
Table S19	Expected annual rates of change in age- and sex-specific population sizes.	20
Figure S1	Observed and expected mortality across age- and sex-specific strata.	21
Figure S2	Association of excess deaths with specific ward-level deprivation indicators	22

Table S1: Excess deaths, relative to continuation of 2018-19 pattern, during control period (1 March to 23 March, 2020).

Stratum	Age group	Predicted (95% UI)	Observed	Excess deaths (95% UI)	Excess mortality ratio (95% UI)
All deaths					
	0-9 years	9 (3, 23)	9	0 (-14, 6)	1.05 (0.38, 3.39)
	10-19 years	5 (2, 14)	11	6 (-3, 9)	2.09 (0.77, 6.34)
	20-29 years	23 (10, 50)	14	-9 (-36, 4)	0.61 (0.28, 1.45)
	30-39 years	25 (14, 42)	36	11 (-6, 22)	1.45 (0.85, 2.52)
	40-49 years	61 (39, 92)	53	-8 (-39, 14)	0.87 (0.58, 1.35)
	50-59 years	97 (70, 133)	92	-5 (-41, 22)	0.95 (0.69, 1.32)
	60-69 years	150 (115, 194)	138	-12 (-56, 23)	0.92 (0.71, 1.20)
	70-79 years	161 (127, 204)	175	14 (-29, 48)	1.09 (0.86, 1.38)
	≥80 years	102 (77, 134)	135	33 (1, 58)	1.32 (1.00, 1.75)
	**All ages**	**624 (552, 704)**	**663**	**39 (-41, 111)**	**1.06 (0.94, 1.20)**
Male deaths					
	0-9 years	5 (1, 18)	5	0 (-13, 4)	1.09 (0.28, 6.06)
	10-19 years	3 (1, 10)	8	5 (-2, 7)	2.83 (0.81, 11.35)
	20-29 years	15 (4, 41)	8	-7 (-33, 4)	0.54 (0.20, 1.80)
	30-39 years	16 (8, 30)	26	10 (-4, 18)	1.64 (0.87, 3.21)
	40-49 years	35 (20, 60)	33	-2 (-27, 13)	0.93 (0.55, 1.63)
	50-59 years	58 (38, 87)	56	-2 (-31, 18)	0.96 (0.64, 1.47)
	60-69 years	87 (61, 122)	80	-7 (-42, 19)	0.92 (0.65, 1.31)
	70-79 years	87 (64, 118)	108	21 (-10, 44)	1.25 (0.92, 1.70)
	≥80 years	51 (34, 75)	69	18 (-6, 35)	1.36 (0.92, 2.01)
	**All ages**	**353 (301, 413)**	**393**	**40 (-20, 92)**	**1.11 (0.95, 1.31)**
Female deaths					
	0-9 years	4 (0, 16)	4	0 (-12, 4)	1.11 (0.25, 9.75)
	10-19 years	2 (0, 13)	3	1 (-10, 3)	1.38 (0.23, ∞)
	20-29 years	8 (2, 25)	6	-2 (-19, 4)	0.79 (0.24, 3.54)
	30-39 years	9 (3, 23)	10	1 (-13, 7)	1.15 (0.44, 3.46)
	40-49 years	25 (12, 48)	20	-5 (-28, 8)	0.81 (0.42, 1.67)
	50-59 years	38 (22, 63)	36	-2 (-27, 14)	0.94 (0.57, 1.61)
	60-69 years	63 (41, 93)	58	-5 (-35, 17)	0.93 (0.62, 1.41)
	70-79 years	74 (50, 107)	67	-7 (-40, 17)	0.91 (0.62, 1.33)
	≥80 years	51 (34, 75)	66	15 (-9, 32)	1.29 (0.88, 1.94)
	**All ages**	**270 (223, 325)**	**270**	**0 (-55, 47)**	**1.00 (0.83, 1.21)**
Deaths by setting					
	Facility	197 (158, 244)	206	9 (-38, 48)	1.05 (0.84, 1.31)
	Community	426 (368, 493)	457	31 (-36, 89)	1.07 (0.93, 1.24)

UI: Uncertainty interval.
Excess deaths are estimated via the difference between observed deaths during 2020-21 and expected deaths for the same periods based on observations in 2018-19, accounting for projected changes in population size. Excess mortality ratios and expected mortality in the pandemic period are computed via Poisson regression models fitted to pre-pandemic (2018-19) and pandemic period (2020-21) observations, accounting for expected changes in population sizes (**Table S17**) via log offset terms. Bold text indicates totals across rows.

Table S2: Excess mortality by healthcare facility or community setting.

Period	Setting	Predicted (95% UI)	Observed	Excess deaths (95% UI)	Excess mortality ratio (95% UI)
Total period (1 March 2020–31 July, 2021)					
	Facility	5147 (4917, 5387)	5866	719 (479, 949)	1.14 (1.09, 1.19)
	Community	10228 (9923, 10543)	14138	3910 (3595, 4215)	1.38 (1.34, 1.42)
Early lockdown (24 March–31 May, 2020)					
	Facility	667 (578, 768)	445	-222 (-323, -133)	0.67 (0.58, 0.77)
	Community	1342 (1235, 1457)	1423	81 (-34, 188)	1.06 (0.98, 1.15)
Wave 1 (1 June–30 September, 2020)					
	Facility	1107 (1013, 1211)	1230	123 (19, 217)	1.11 (1.02, 1.21)
	Community	2207 (2091, 2329)	3718	1511 (1389, 1627)	1.68 (1.60, 1.78)
Wave 2 (16 March–15 July, 2021)					
	Facility	1141 (1061, 1225)	2225	1084 (1000, 1164)	1.95 (1.82, 2.10)
	Community	2309 (2193, 2432)	4160	1851 (1728, 1967)	1.80 (1.71, 1.90)

UI: Uncertainty interval.

Excess deaths are estimated via the difference between observed deaths during 2020-21 and expected deaths for the same periods based on observations in 2018-19, accounting for projected changes in population size. Excess mortality ratios and expected mortality in the pandemic period are computed via Poisson regression models fitted to pre-pandemic (2018-19) and pandemic period (2020-21) observations, accounting for expected changes in population sizes (**Table S17**) via log offset terms.

Table S3: Excess deaths, relative to continuation of 2018-19 pattern—Males.

Period	Age group	Predicted (95% UI)	Observed	Excess deaths (95% UI)	Excess mortality ratio (95% UI)
Total period (1 March 2020–31 July, 2021)					
	0-9 years	135 (95, 190)	85	-50 (-105, -10)	0.63 (0.45, 0.89)
	10-19 years	121 (83, 173)	75	-46 (-98, -8)	0.62 (0.43, 0.90)
	20-29 years	278 (223, 345)	229	-49 (-116, 6)	0.82 (0.66, 1.02)
	30-39 years	447 (382, 522)	484	37 (-38, 102)	1.08 (0.93, 1.27)
	40-49 years	969 (873, 1,075)	1,121	152 (46, 248)	1.16 (1.04, 1.28)
	50-59 years	1,636 (1,511, 1,771)	1,999	363 (228, 488)	1.22 (1.13, 1.32)
	60-69 years	2,192 (2,049, 2,345)	2,816	624 (471, 767)	1.28 (1.20, 1.37)
	70-79 years	2,035 (1,904, 2,175)	3,076	1,041 (901, 1,172)	1.51 (1.41, 1.62)
	≥80 years	1,436 (1,324, 1,556)	2,039	603 (483, 715)	1.42 (1.31, 1.54)
	**All ages**	**9,091 (8,797, 9,395)**	**11,924**	**2,833 (2,529, 3,127)**	**1.31 (1.27, 1.36)**
Early lockdown (24 March–31 May, 2020)					
	0-9 years	25 (5, 84)	5	-20 (-79, 0)	0.20 (0.06, 1.03)
	10-19 years	17 (5, 45)	8	-9 (-37, 3)	0.48 (0.18, 1.60)
	20-29 years	28 (13, 53)	20	-8 (-33, 7)	0.72 (0.37, 1.49)
	30-39 years	67 (42, 104)	45	-22 (-59, 3)	0.67 (0.43, 1.06)
	40-49 years	125 (92, 167)	107	-18 (-60, 15)	0.86 (0.64, 1.16)
	50-59 years	213 (167, 269)	164	-49 (-105, -3)	0.77 (0.61, 0.98)
	60-69 years	284 (232, 344)	239	-45 (-105, 7)	0.84 (0.69, 1.03)
	70-79 years	259 (212, 316)	240	-19 (-76, 28)	0.93 (0.76, 1.13)
	≥80 years	184 (146, 229)	194	10 (-35, 48)	1.06 (0.85, 1.33)
	**All ages**	**1,184 (1,075, 1,304)**	**1022**	**-162 (-282, -53)**	**0.86 (0.78, 0.95)**
Wave 1 (1 June–30 September, 2020)					
	0-9 years	25 (13, 48)	21	-4 (-27, 8)	0.83 (0.43, 1.67)
	10-19 years	30 (13, 62)	15	-15 (-47, 2)	0.51 (0.24, 1.15)
	20-29 years	70 (46, 103)	58	-12 (-45, 12)	0.83 (0.56, 1.25)
	30-39 years	103 (76, 138)	115	12 (-23, 39)	1.12 (0.84, 1.51)
	40-49 years	225 (183, 274)	246	21 (-28, 63)	1.10 (0.90, 1.34)
	50-59 years	368 (318, 426)	474	106 (48, 156)	1.29 (1.11, 1.49)
	60-69 years	477 (422, 539)	720	243 (181, 298)	1.51 (1.34, 1.71)
	70-79 years	430 (382, 484)	832	402 (348, 450)	1.93 (1.72, 2.18)
	≥80 years	291 (252, 336)	574	283 (238, 322)	1.97 (1.71, 2.28)
	**All ages**	**1,988 (1,874, 2,108)**	**3055**	**1,067 (947, 1,181)**	**1.54 (1.45, 1.63)**
Wave 2 (16 March–15 July, 2021)					
	0-9 years	38 (17, 76)	16	-22 (-60, -1)	0.42 (0.21, 0.94)
	10-19 years	26 (10, 59)	12	-14 (-47, 2)	0.46 (0.20, 1.17)
	20-29 years	61 (40, 92)	54	-7 (-38, 14)	0.89 (0.59, 1.36)
	30-39 years	116 (87, 151)	133	17 (-18, 46)	1.15 (0.88, 1.52)
	40-49 years	220 (184, 261)	366	146 (105, 182)	1.67 (1.40, 1.99)
	50-59 years	368 (322, 419)	660	292 (241, 338)	1.79 (1.58, 2.05)
	60-69 years	480 (428, 536)	902	422 (366, 474)	1.88 (1.68, 2.11)
	70-79 years	445 (399, 497)	1014	569 (517, 615)	2.28 (2.04, 2.54)
	≥80 years	307 (268, 352)	634	327 (282, 366)	2.06 (1.80, 2.37)
	**All ages**	**2,028 (1,920, 2,142)**	**3791**	**1,763 (1,649, 1,871)**	**1.87 (1.77, 1.97)**

UI: Uncertainty interval.

Excess deaths are estimated via the difference between observed deaths during 2020-21 and expected deaths for the same periods based on observations in 2018-19, accounting for projected changes in population size. Excess mortality ratios and expected mortality in the pandemic period are computed via Poisson regression models fitted to pre-pandemic (2018-19) and pandemic period (2020-21) observations, accounting for expected changes in population sizes (**Table S17**) via log offset terms. Bold text indicates totals across rows.

Table S4: Excess deaths, relative to continuation of 2018-19 pattern—Females.

Period	Age group	Predicted (95% UI)	Observed	Excess deaths (95% UI)	Excess mortality ratio (95% UI)
Total period (1 March 2020–31 July, 2021)					
	0-9 years	100 (68, 144)	77	-23 (-67, 9)	0.77 (0.53, 1.13)
	10-19 years	55 (35, 86)	59	4 (-27, 24)	1.07 (0.69, 1.68)
	20-29 years	133 (98, 179)	127	-6 (-52, 29)	0.95 (0.71, 1.29)
	30-39 years	214 (168, 270)	207	-7 (-63, 39)	0.97 (0.77, 1.23)
	40-49 years	484 (416, 563)	521	37 (-42, 105)	1.08 (0.93, 1.25)
	50-59 years	920 (829, 1,021)	1,173	253 (152, 344)	1.27 (1.15, 1.41)
	60-69 years	1,483 (1,365, 1,609)	1,876	393 (267, 511)	1.27 (1.17, 1.37)
	70-79 years	1,697 (1,573, 1,830)	2,289	592 (459, 716)	1.35 (1.25, 1.46)
	≥80 years	1,327 (1,216, 1,446)	1,751	424 (305, 535)	1.32 (1.21, 1.44)
	**All ages**	**6,285 (6,040, 6,538)**	**8,080**	**1,795 (1,542, 2,040)**	**1.29 (1.24, 1.34)**
Early lockdown (24 March–31 May, 2020)					
	0-9 years	12 (5, 27)	14	2 (-13, 9)	1.16 (0.51, 2.86)
	10-19 years	8 (2, 27)	6	-2 (-21, 4)	0.71 (0.22, 3.20)
	20-29 years	16 (6, 36)	13	-3 (-23, 7)	0.81 (0.36, 2.02)
	30-39 years	30 (15, 57)	20	-10 (-37, 5)	0.68 (0.35, 1.38)
	40-49 years	65 (40, 101)	43	-22 (-58, 3)	0.67 (0.43, 1.07)
	50-59 years	120 (91, 157)	133	13 (-24, 42)	1.11 (0.85, 1.46)
	60-69 years	189 (150, 238)	184	-5 (-54, 34)	0.97 (0.77, 1.22)
	70-79 years	219 (177, 269)	220	1 (-49, 43)	1.00 (0.82, 1.24)
	≥80 years	179 (144, 222)	213	34 (-9, 69)	1.19 (0.96, 1.48)
	**All ages**	**825 (740, 918)**	**846**	**21 (-72, 106)**	**1.03 (0.92, 1.14)**
Wave 1 (1 June–30 September, 2020)					
	0-9 years	23 (9, 53)	12	-11 (-41, 3)	0.52 (0.23, 1.33)
	10-19 years	12 (3, 35)	7	-5 (-28, 4)	0.59 (0.20, 2.22)
	20-29 years	23 (12, 41)	27	4 (-14, 15)	1.19 (0.66, 2.24)
	30-39 years	43 (27, 68)	47	4 (-21, 20)	1.08 (0.69, 1.74)
	40-49 years	107 (80, 141)	126	19 (-15, 46)	1.18 (0.89, 1.57)
	50-59 years	199 (162, 243)	249	50 (6, 87)	1.25 (1.02, 1.53)
	60-69 years	313 (268, 364)	450	137 (86, 182)	1.44 (1.24, 1.68)
	70-79 years	358 (311, 411)	554	196 (143, 243)	1.55 (1.35, 1.78)
	≥80 years	273 (232, 320)	421	148 (101, 189)	1.54 (1.32, 1.81)
	**All ages**	**1,326 (1,231, 1,429)**	**1,893**	**567 (464, 662)**	**1.43 (1.33, 1.54)**
Wave 2 (16 March–15 July, 2021)					
	0-9 years	22 (10, 44)	17	-5 (-27, 7)	0.78 (0.38, 1.71)
	10-19 years	13 (6, 27)	20	7 (-7, 14)	1.52 (0.75, 3.25)
	20-29 years	29 (17, 48)	37	8 (-11, 20)	1.28 (0.77, 2.22)
	30-39 years	52 (35, 77)	65	13 (-12, 30)	1.25 (0.85, 1.87)
	40-49 years	109 (85, 139)	172	63 (33, 87)	1.58 (1.23, 2.04)
	50-59 years	204 (171, 242)	388	184 (146, 217)	1.90 (1.61, 2.26)
	60-69 years	328 (286, 376)	606	278 (230, 320)	1.85 (1.61, 2.12)
	70-79 years	379 (335, 428)	783	404 (355, 448)	2.07 (1.83, 2.34)
	≥80 years	313 (270, 362)	506	193 (144, 236)	1.61 (1.40, 1.87)
	**All ages**	**1,421 (1,331, 1,518)**	**2,594**	**1,173 (1,076, 1,263)**	**1.82 (1.71, 1.95)**

UI: Uncertainty interval.

Excess deaths are estimated via the difference between observed deaths during 2020-21 and expected deaths for the same periods based on observations in 2018-19, accounting for projected changes in population size. Excess mortality ratios and expected mortality in the pandemic period are computed via Poisson regression models fitted to pre-pandemic (2018-19) and pandemic period (2020-21) observations, accounting for expected changes in population sizes (**Table S17**) via log offset terms. Bold text indicates totals across rows.

Table S5: Comparison of attributed causes of death during the pre-pandemic period to verbal autopsy-based estimates for all of India, 2017-2019, by age and sex.

Age group	Attributed cause	All, %		Males, %		Females, %	
		Observed	SRS 2017-19	Observed	SRS 2017-19	Observed	SRS 2017-19
0-4y							
	Infections (other than COVID-19), perinatal, and maternal diseases	25.0	83.6	24.4	82.6	25.7	84.7
	Noncommunicable diseases	21.9	10.1	22.2	10.7	21.4	9.5
	Injuries	5.7	4.9	6.7	5.1	4.3	4.7
	Uncategorized (including senility)	47.5	1.4	46.7	1.6	48.6	1.2
	**Proportion of all deaths belonging to age group**	**1.0**	**4.3**				
5-14y							
	Infections (other than COVID-19), perinatal, and maternal diseases	18.6	38.2	18.8	34.4	18.4	42.8
	Noncommunicable diseases	29.5	25.0	28.8	23.1	30.6	27.1
	Injuries	11.6	33.5	10.0	39.6	14.3	26.3
	Uncategorized (including senility)	40.3	3.3	42.5	2.9	36.7	3.7
	**Proportion of all deaths belonging to age group**	**0.8**	**0.8**				
15-29y							
	Infections (other than COVID-19), perinatal, and maternal diseases	4.7	19.8	3.8	14.0	6.4	28.9
	Noncommunicable diseases	27.2	29.5	27.3	28.1	26.9	31.5
	Injuries	44.3	47.1	48.1	54.7	36.3	35.3
	Uncategorized (including senility)	23.8	3.6	20.8	3.2	30.4	4.2
	**Proportion of all deaths belonging to age group**	**3.5**	**5.5**				
30-44y							
	Infections (other than COVID-19), perinatal, and maternal diseases	3.8	14.3	3.5	12.1	4.3	19.5
	Noncommunicable diseases	57.6	55.4	56.2	54.0	60.5	58.6
	Injuries	16.1	28.0	19.2	31.9	9.6	19.1
	Uncategorized (including senility)	22.6	2.3	21.2	2.0	25.5	2.8
	**Proportion of all deaths belonging to age group**	**8.4**	**2.4**				
45-54y							
	Infections (other than COVID-19), perinatal, and maternal diseases	3.7	12.2	3.7	11.1	3.8	14.4
	Noncommunicable diseases	71.6	72.7	69.9	71.9	74.7	74.3
	Injuries	5.8	12.9	7.4	15.0	2.9	8.5
	Uncategorized (including senility)	18.9	2.2	19.0	1.9	18.6	2.7
	**Proportion of all deaths belonging to age group**	**12.5**	**6.6**				
55-69y							
	Infections (other than COVID-19), perinatal, and maternal diseases	2.9	14.7	2.7	13.5	3.2	16.6
	Noncommunicable diseases	76.2	74.4	75.5	75.5	77.2	72.6
	Injuries	2.6	6.1	3.2	6.9	1.6	4.9
	Uncategorized (including senility)	18.4	4.8	18.7	4.0	18.0	5.9
	**Proportion of all deaths belonging to age group**	**32.6**	**10.6**				
≥70y							
	Infections (other than COVID-19), perinatal, and maternal diseases	2.7	18.1	2.6	17.3	2.7	19.0
	Noncommunicable diseases	75.8	52.5	76.0	56.9	75.7	47.9
	Injuries	1.2	3.7	1.5	3.6	0.9	3.8
	Uncategorized (including senility)	20.3	25.7	20.0	22.3	20.7	29.3
	**Proportion of all deaths belonging to age group**	**41.1**	**40.9**				
All							
	Infections (other than COVID-19), perinatal, and maternal diseases	3.4	21.5	3.3	19.4	3.6	24.3
	Noncommunicable diseases	71.2	56.0	70.1	58.3	72.8	52.7
	Injuries	5.1	10.4	6.6	12.5	3.0	7.5
	Uncategorized (including senility)	20.3	12.2	20.0	9.7	20.7	15.5

SRS: Sample-based registration system.

SRS data based on verbal autopsies are obtained from ref. 34, India Sample-Based Registration System Bulletin 2020. Available from: https://censusindia.gov.in/nada/index.php/catalog/42687. Accessed 22 March, 2023. Bold text indicates totals across rows.

Table S6: Comparison of attributed causes of death during the pre-pandemic period to verbal autopsy-based estimates, for adults aged 15-69 years in low-mortality districts, 2014.

Age group	Attributed cause	All, %		Males, %		Females, %	
		Observed	Low-mortality districts	Observed	Low-mortality districts	Observed	Low-mortality districts
All 15-69							
	Infections (other than COVID-19), perinatal, and maternal diseases	3.3	19.4	3.1	19.1	3.6	19.9
	Noncommunicable diseases	69.4	60.5	68.0	59.8	71.8	61.5
	Injuries	7.8	15.5	9.7	17.3	4.8	12.7
	Uncategorized (including senility)	19.4	4.6	19.3	3.8	19.8	6.0

Data on attributed causes of death across low-mortality districts based on verbal autopsies are obtained from ref. 35, Ram, U. *et al.* Age-specific and sex-specific adult mortality risk in India in 2014: analysis of 0·27 million nationally surveyed deaths and demographic estimates from 597 districts. *Lancet Global Health* **3**, e767–e775 (2015).

Table S7: Excess deaths by attributed cause—Control period (1 March–23 March, 2020).

Attributed cause	Predicted (95% UI)	Observed	Excess deaths (95% UI)	Excess mortality ratio (95% UI)
Infections (other than COVID-19) and maternal diseases	21 (0, 100)	3	-18 (-97, 3)	0.14 (0.03, ∞)
Noncommunicable diseases	435 (377, 499)	503	68 (4, 126)	1.16 (1.01, 1.33)
Injuries	35 (21, 58)	38	3 (-20, 17)	1.07 (0.65, 1.81)
Senility	21 (11, 38)	28	7 (-10, 17)	1.31 (0.73, 2.46)
Other unclassified causes	108 (78, 147)	91	-17 (-56, 13)	0.85 (0.62, 1.17)

UI: Uncertainty interval.

Excess deaths are estimated via the difference between observed deaths during 2020-21 and expected deaths for the same periods based on observations in 2018-19, accounting for projected changes in population size. Excess mortality ratios and expected mortality in the pandemic period are computed via Poisson regression models fitted to pre-pandemic (2018-19) and pandemic period (2020-21) observations, accounting for expected changes in population sizes (**Table S17**) via log offset terms.

Table S8: Excess deaths by attributed cause— Early lockdown (24 March–31 May, 2020).

Cause		Predicted (95% UI)	Observed	Excess deaths (95% UI)	Excess mortality ratio (95% UI)
Infections (other than COVID-19) and maternal diseases					
	Lower respiratory tract infections	32 (10, 85)	8	-24 (-77, -2)	0.25 (0.09, 0.81)
	Tuberculosis	6 (1, 21)	5	-1 (-16, 4)	0.89 (0.24, 4.87)
	Diarrhea and gastrointestinal infections	3 (0, 15)	3	0 (-12, 3)	1.13 (0.20, ∞)
	Syphilis and other genitourinary diseases	3 (0, 22)	2	-1 (-20, 2)	0.69 (0.09, ∞)
	Other infectious diseases	0 (0, 0)	0	0 (0, 0)	--
	Maternal and perinatal diseases	1 (0, 19)	1	0 (-18, 1)	1.26 (0.05, ∞)
	**Total**	**49 (24, 92)**	**19**	**-30 (-73, -5)**	**0.39 (0.21, 0.81)**
Noncommunicable diseases					
	Cardiovascular/cerebrovascular conditions	1,146 (1,047, 1,252)	1,221	75 (-31, 174)	1.07 (0.97, 1.17)
	Chronic liver disease	57 (25, 120)	14	-43 (-106, -11)	0.24 (0.12, 0.57)
	Cancer	89 (69, 114)	186	97 (72, 117)	2.09 (1.63, 2.69)
	Diabetes mellitus	61 (40, 91)	56	-5 (-35, 16)	0.92 (0.62, 1.41)
	Other noncommunicable diseases	55 (11, 183)	5	-50 (-178, -6)	0.09 (0.03, 0.46)
	**Total**	**1,415 (1,305, 1,534)**	**1,482**	**67 (-52, 177)**	**1.05 (0.97, 1.14)**
Injuries					
	Unintentional	83 (49, 137)	33	-50 (-104, -16)	0.40 (0.24, 0.67)
	Intentional (suicide, homicide)	30 (16, 55)	24	-6 (-31, 8)	0.80 (0.44, 1.54)
	**Total**	**114 (76, 167)**	**57**	**-57 (-110, -19)**	**0.50 (0.34, 0.75)**
Other or unclassified causes					
	Senility	54 (38, 77)	81	27 (4, 43)	1.49 (1.05, 2.15)
	Other unclassified causes	376 (308, 457)	229	-147 (-228, -79)	0.61 (0.50, 0.74)
	COVID-19	0	0	– –	– –

UI: Uncertainty interval.
Excess deaths are estimated via the difference between observed deaths during 2020-21 and expected deaths for the same periods based on observations in 2018-19, accounting for projected changes in population size. Excess mortality ratios and expected mortality in the pandemic period are computed via Poisson regression models fitted to pre-pandemic (2018-19) and pandemic period (2020-21) observations, accounting for expected changes in population sizes (**Table S17**) via log offset terms. Bold text indicates totals across rows.

Table S9: Excess deaths by attributed cause—Wave 1 (1 June–30 September, 2020).

Cause		Predicted (95% UI)	Observed	Excess deaths (95% UI)	Excess mortality ratio (95% UI)
Infections (other than COVID-19) and maternal diseases					
	Lower respiratory tract infections	49 (28, 84)	29	-20 (-55, 1)	0.59 (0.34, 1.05)
	Tuberculosis	10 (3, 30)	7	-3 (-23, 4)	0.69 (0.23, 2.66)
	Diarrhea and gastrointestinal infections	8 (2, 30)	5	-3 (-25, 3)	0.59 (0.16, 3.19)
	Syphilis and other genitourinary diseases	6 (2, 19)	7	1 (-12, 5)	1.12 (0.36, 4.40)
	Other infectious diseases	0 (0, 0)	0	0 (0, 0)	– –
	Maternal and perinatal diseases	0 (0, 10)	1	1 (-9, 1)	4.64 (0.10, ∞)
	**Total**	**85 (55, 129)**	**49**	**-36 (-80, -6)**	**0.58 (0.38, 0.89)**
Noncommunicable diseases					
	Cardiovascular/cerebrovascular conditions	2009 (1895, 2128)	3203	1194 (1075, 1308)	1.59 (1.51, 1.69)
	Chronic liver disease	117 (72, 184)	38	-79 (-146, -34)	0.32 (0.21, 0.53)
	Cancer	236 (196, 285)	284	48 (-1, 88)	1.20 (1.00, 1.45)
	Diabetes mellitus	82 (62, 108)	145	63 (37, 83)	1.76 (1.34, 2.33)
	Other noncommunicable diseases	25 (9, 58)	11	-14 (-47, 2)	0.44 (0.19, 1.18)
	**Total**	2,472 (2,344, 2,607)	3,681	1,209 (1,074, 1,337)	1.49 (1.41, 1.57)
Injuries and accidents					
	Unintentional	110 (78, 153)	79	-31 (-74, 1)	0.72 (0.51, 1.02)
	Intentional (suicide, homicide)	59 (41, 85)	76	17 (-9, 35)	1.28 (0.89, 1.87)
	**Total**	**170 (132, 217)**	**155**	**-15 (-62, 23)**	**0.91 (0.72, 1.17)**
Other or unclassified causes					
	Senility	86 (67, 109)	211	125 (102, 144)	2.47 (1.94, 3.14)
	Other unclassified causes	501 (436, 574)	525	24 (-49, 89)	1.05 (0.92, 1.20)
	COVID-19	0	327	327	– –

UI: Uncertainty interval.
Excess deaths are estimated via the difference between observed deaths during 2020-21 and expected deaths for the same periods based on observations in 2018-19, accounting for projected changes in population size. Excess mortality ratios and expected mortality in the pandemic period are computed via Poisson regression models fitted to pre-pandemic (2018-19) and pandemic period (2020-21) observations, accounting for expected changes in population sizes (**Table S17**) via log offset terms. Bold text indicates totals across rows.

Table S10: Excess deaths by attributed cause—Wave 2 (16 March–15 July, 2021).

Attributed cause		Predicted (95% UI)	Observed	Excess deaths (95% UI)	Excess mortality ratio (95% UI)
Infections (other than COVID-19) and maternal diseases					
	Lower respiratory tract infections	60 (43, 84)	101	41 (17, 58)	1.67 (1.21, 2.34)
	Tuberculosis	10 (4, 25)	12	2 (-13, 8)	1.17 (0.48, 3.15)
	Diarrhea and gastrointestinal infections	6 (0, 41)	2	-4 (-39, 2)	0.34 (0.05, ∞)
	Syphilis and other genitourinary diseases	7 (1, 29)	4	-3 (-25, 3)	0.58 (0.14, 4.55)
	Other infectious diseases	0 (0, 0)	0	0 (0, 0)	– –
	Maternal and perinatal diseases	0 (0, 0)	0	0 (0, 0)	– –
	**Total**	**93 (69, 124)**	**119**	**26 (-5, 50)**	**1.29 (0.96, 1.73)**
Noncommunicable diseases					
	Cardiovascular/cerebrovascular conditions	2015 (1895, 2143)	2720	705 (577, 825)	1.35 (1.27, 1.44)
	Chronic liver disease	105 (66, 162)	43	-62 (-119, -23)	0.41 (0.27, 0.65)
	Cancer	177 (132, 237)	101	-76 (-136, -31)	0.57 (0.43, 0.77)
	Diabetes mellitus	103 (77, 135)	127	24 (-8, 50)	1.24 (0.94, 1.65)
	Other noncommunicable diseases	76 (29, 172)	11	-65 (-161, -18)	0.14 (0.06, 0.37)
	**Total**	**2,480 (2,342, 2,628)**	**3,002**	**522 (374, 660)**	**1.21 (1.14, 1.28)**
Injuries					
	Unintentional	140 (94, 203)	57	-83 (-146, -37)	0.41 (0.28, 0.61)
	Intentional (suicide, homicide)	56 (37, 82)	66	10 (-16, 29)	1.19 (0.81, 1.76)
	**Total**	**196 (149, 255)**	**123**	**-73 (-132, -26)**	**0.63 (0.48, 0.83)**
Other or unclassified causes					
	Senility	96 (75, 121)	208	112 (87, 133)	2.17 (1.71, 2.77)
	Other unclassified causes	584 (537, 635)	2127	1543 (1492, 1590)	3.64 (3.35, 3.96)
	COVID-19	0	806	806	– –

UI: Uncertainty interval.

Excess deaths are estimated via the difference between observed deaths during 2020-21 and expected deaths for the same periods based on observations in 2018-19, accounting for projected changes in population size. Excess mortality ratios and expected mortality in the pandemic period are computed via Poisson regression models fitted to pre-pandemic (2018-19) and pandemic period (2020-21) observations, accounting for expected changes in population sizes (**Table S17**) via log offset terms. Bold text indicates totals across rows.

Table S11: Excess deaths by attributed cause—Deaths occurring in healthcare facilities during the total pandemic period (1 March, 2020–31 July, 2021).

Attributed cause		Predicted (95% UI)	Observed	Excess deaths (95% UI)	Excess mortality ratio (95% UI)
Infections (other than COVID-19) and maternal diseases					
	Lower respiratory tract infections	204 (155, 267)	142	-62 (-125, -13)	0.69 (0.53, 0.92)
	Tuberculosis	29 (11, 71)	11	-18 (-60, 0)	0.38 (0.16, 1.03)
	Diarrhea and gastrointestinal infections	1 (0, 29)	1	0 (-28, 1)	1.02 (0.03, ∞)
	Syphilis and other genitourinary diseases	6 (1, 30)	4	-2 (-26, 3)	0.63 (0.14, 5.76)
	Other infectious diseases	0 (0, 0)	0	0 (0, 0)	--
	Maternal and perinatal diseases	2 (0, 23)	2	0 (-21, 2)	0.82 (0.09, ∞)
	**Total**	**254 (197, 325)**	**160**	**-94 (-165, -37)**	**0.63 (0.49, 0.81)**
Noncommunicable diseases					
	Cardiovascular/cerebrovascular conditions	2,098 (1,946, 2,263)	1,997	-101 (-266, 51)	0.95 (0.88, 1.03)
	Chronic liver disease	123 (86, 173)	85	-38 (-88, -1)	0.69 (0.49, 0.99)
	Cancer	271 (220, 333)	273	2 (-60, 53)	1.01 (0.82, 1.24)
	Diabetes mellitus	174 (134, 225)	175	1 (-50, 41)	1.00 (0.78, 1.30)
	Other noncommunicable diseases	28 (14, 53)	28	0 (-25, 14)	1.00 (0.53, 1.96)
	**Total**	**2,696 (2,520, 2,881)**	**2,558**	**-138 (-323, 38)**	**0.95 (0.89, 1.02)**
Injuries					
	Unintentional	313 (245, 397)	168	-145 (-229, -77)	0.54 (0.42, 0.69)
	Intentional (suicide, homicide)	7 (1, 25)	6	-1 (-19, 5)	0.91 (0.24, 4.50)
	**Total**	**320 (251, 405)**	**174**	**-146 (-231, -77)**	**0.54 (0.43, 0.69)**
Other or unclassified causes					
	Senility	22 (0, 107)	3	-19 (-104, 3)	0.13 (0.03, ∞)
	Other unclassified causes	1,851 (1,708, 2,005)	1,785	-66 (-220, 77)	0.96 (0.89, 1.04)
	COVID-19	– –	1,186	1,186	– –

UI: Uncertainty interval.

Excess deaths are estimated via the difference between observed deaths during 2020-21 and expected deaths for the same periods based on observations in 2018-19, accounting for projected changes in population size. Excess mortality ratios and expected mortality in the pandemic period are computed via Poisson regression models fitted to pre-pandemic (2018-19) and pandemic period (2020-21) observations, accounting for expected changes in population sizes (**Table S17**) via log offset terms. Bold text indicates totals across rows.

Table S12: Excess deaths by attributed cause—Deaths occurring in the community during the total pandemic period (1 March, 2020–31 July, 2021).

Attributed cause		Predicted (95% UI)	Observed	Excess deaths (95% UI)	Excess mortality ratio (95% UI)
Infections (other than COVID-19) and maternal diseases					
	Lower respiratory tract infections	148 (89, 239)	36	-112 (-203, -53)	0.24 (0.15, 0.41)
	Tuberculosis	18 (9, 35)	29	11 (-6, 20)	1.62 (0.82, 3.30)
	Diarrhea and gastrointestinal infections	42 (15, 102)	10	-32 (-92, -5)	0.24 (0.10, 0.68)
	Syphilis and other genitourinary diseases	21 (10, 42)	23	2 (-19, 13)	1.10 (0.55, 2.35)
	Other infectious diseases	24 (0, 158)	2	-22 (-156, 2)	0.09 (0.01, ∞)
	Maternal and perinatal diseases	0 (0, 0)	0	0 (0, 0)	--
	**Total**	**265 (195, 357)**	**100**	**-165 (-257, -95)**	**0.38 (0.28, 0.51)**
Noncommunicable diseases					
	Cardiovascular/cerebrovascular conditions	6,754 (6,510, 7,009)	9,701	2,947 (2,692, 3,191)	1.44 (1.38, 1.49)
	Chronic liver disease	332 (245, 446)	98	-234 (-348, -147)	0.30 (0.22, 0.40)
	Cancer	584 (505, 674)	534	-50 (-140, 29)	0.91 (0.79, 1.06)
	Diabetes mellitus	263 (217, 318)	362	99 (44, 145)	1.38 (1.14, 1.67)
	Other noncommunicable diseases	314 (172, 545)	25	-289 (-520, -147)	0.08 (0.05, 0.15)
	**Total**	**8,254 (7,974, 8,543)**	**10,720**	**2,466 (2,177, 2,746)**	**1.30 (1.25, 1.34)**
Injuries					
	Unintentional	209 (162, 267)	174	-35 (-93, 12)	0.83 (0.65, 1.08)
	Intentional (suicide, homicide)	258 (210, 315)	295	37 (-20, 85)	1.14 (0.94, 1.40)
	**Total**	**467 (398, 546)**	**469**	**2 (-77, 71)**	**1.01 (0.86, 1.18)**
Other or unclassified causes					
	Senility	397 (345, 457)	776	379 (319, 431)	1.95 (1.70, 2.25)
	Other unclassified causes	843 (770, 924)	2,019	1,176 (1,095, 1,249)	2.39 (2.18, 2.62)
	COVID-19	– –	54	54	– –

UI: Uncertainty interval.
Excess deaths are estimated via the difference between observed deaths during 2020-21 and expected deaths for the same periods based on observations in 2018-19, accounting for projected changes in population size. Excess mortality ratios and expected mortality in the pandemic period are computed via Poisson regression models fitted to pre-pandemic (2018-19) and pandemic period (2020-21) observations, accounting for expected changes in population sizes (**Table S17**) via log offset terms. Bold text indicates totals across rows.

Table S13: Reported causes of death among individuals with or without documented SARS-CoV-2 infection.

Attributed cause		Decedent classification		
		2020-21 decedents with documented infection, *n* (%)	2020-21 decedents without documented infection, *n* (%)	2018-19 reference period decedents, *n* (%)
		N=578	*N*=19,426	*N*=15,239
Infections (other than COVID-19) and maternal diseases				
	Lower respiratory tract infections	5 (0.9)	173 (0.9)	351 (2.3)
	Tuberculosis	0	40 (0.2)	48 (0.3)
	Diarrhea and gastrointestinal infections	0	11 (0.1)	45 (0.3)
	Syphilis and other genitourinary diseases	0	27 (0.1)	28 (0.2)
	Other infectious diseases	0	2 (<0.1)	35 (0.2)
	Maternal and perinatal diseases	0	2 (<0.1)	9 (0.1)
	**Total**	**5 (0.9)**	**255 (1.3)**	**516 (3.4)**
Noncommunicable diseases				
	Cardiovascular/cerebrovascular conditions	105 (18.2)	11,593 (59.7)	8,774 (57.6)
	Chronic liver disease	1 (0.2)	182 (0.9)	452 (3.0)
	Cancer	1 (0.2)	806 (4.1)	848 (5.6)
	Diabetes mellitus	4 (0.7)	533 (2.7)	434 (2.8)
	Other noncommunicable diseases	0 (0.0)	53 (0.3)	344 (2.3)
	**Total**	**111 (19.2)**	**13,167 (67.8)**	**10,852 (71.2)**
Injuries				
	Unintentional	0	342 (1.8)	518 (3.4)
	Intentional (suicide, homicide)	1 (0.2)	300 (1.5)	263 (1.7)
	**Total**	**1 (0.2)**	**642 (3.3)**	**781 (5.1)**
Other or unclassified causes				
	Senility	3 (0.5)	776 (4.0)	419 (2.7)
	Other unclassified causes	79 (13.7)	3,725 (19.2)	2,671 (17.5)
	COVID-19	379 (65.6)	861 (4.4)	– –

UI: Uncertainty interval.
Excess deaths are estimated via the difference between observed deaths during 2020-21 and expected deaths for the same periods based on observations in 2018-19, accounting for projected changes in population size. Excess mortality ratios and expected mortality in the pandemic period are computed via Poisson regression models fitted to pre-pandemic (2018-19) and pandemic period (2020-21) observations, accounting for expected changes in population sizes (**Table S17**) via log offset terms. Bold text indicates totals across rows.

Table S14: Association of ward-level characteristics with deprivation index.

Characteristic	β (95% CI)
Household crowding/density	0.07 (–0.13, 0.27)
Proportion of adults illiterate	0.81 (0.69, 0.93)
Proportion of households inhabiting dilapidated dwelling structures	0.26 (0.07, 0.45)
Proportion of households inhabiting dwellings with unfinished flooring	0.80 (0.68, 0.92)
Proportion of households without access to tapped, treated water sources	0.22 (0.03, 0.42)
Proportion of households without access to onsite water sources	0.87 (0.77, 0.97)
Proportion of households without onsite latrines	0.90 (0.82, 0.98)
Proportion of households without onsite sewer connections	0.80 (0.68, 0.92)
Proportion of households reliant upon open defecation	0.64 (0.49, 0.80)
Proportion of households without improved cooking fuel	0.79 (0.67, 0.91)
Proportion of households lacking bank accounts	0.55 (0.38, 0.71)
Proportion of households lacking any index assets[1]	0.80 (0.69, 0.92)
Proportion of households inhabiting non-permanent dwelling structures	0.75 (0.62, 0.89)
Proportion of households without electric lighting	0.80 (0.68, 0.92)
Proportion of population belonging to scheduled castes or tribes	0.64 (0.49, 0.80)

CI: Confidence interval.

Values indicate the association (estimated via regression) of each attribute with the first principal component; each measure is centered and scaled to unit variance. The first principal component explained 48.1% of variance across all covariates.

[1]Index assets are defined as radios, televisions, in-home internet access via any device, telephones, bicycles, scooters, or cars; households responding "No" for all assets were considered to lack any index asset.

Table S15: Association of cause-specific excess deaths with ward-level deprivation index.

Attributed cause of death		β (95% UI)			
		Total period	*Early lockdown*	*Wave 1*	*Wave 2*
Infections (other than COVID-19) and maternal diseases					
	Lower respiratory tract infections	0.19 (-0.05, 0.42)	- -	0.22 (-0.52, 1.15)	1.04 (-0.13, 2.64)
	**All infections and maternal diseases**	0.25 (0.02, 0.48)	0.08 (-1.16, 1.36)	0.17 (-0.22, 0.65)	0.81 (0.06, 1.83)
Noncommunicable diseases					
	Cardiovascular/cerebrovascular conditions	0.04 (-0.01, 0.09)	0.11 (-0.02, 0.23)	-0.06 (-0.15, 0.04)	0.06 (-0.03, 0.16)
	Chronic liver disease	0.10 (-0.06, 0.25)	0.03 (-0.71, 1.04)	0.01 (-0.39, 0.47)	-0.04 (-0.73, 0.46)
	Cancer	0.01 (-0.15, 0.16)	-0.54 (-1.35, 0.22)	-0.08 (-0.35, 0.22)	0.07 (-0.21, 0.36)
	Diabetes mellitus	-0.02 (-0.28, 0.24)	-0.36 (-1.10, 0.29)	-0.14 (-0.78, 0.37)	0.33 (-0.17, 0.78)
	**All noncommunicable diseases**	**0.06 (0.02, 0.10)**	**0.06 (-0.04, 0.16)**	**-0.03 (-0.12, 0.05)**	**0.11 (0.03, 0.19)**
Injuries					
	Unintentional	0.00 (-0.16, 0.17)	0.08 (-0.63, 0.53)	0.03 (-0.51, 0.46)	0.02 (-0.28, 0.26)
	Intentional (suicide, homicide)	0.02 (-0.20, 0.32)	0.02 (-0.59, 0.72)	-0.03 (-0.51, 0.44)	-0.04 (-0.51, 0.43)
	**Total**	-0.01 (-0.16, 0.15)	0.06 (-0.20, 0.36)	0.01 (-0.32, 0.33)	0.01 (-0.21, 0.22)

UI: Uncertainty interval.
Excess deaths are estimated via the difference between observed deaths during 2020-21 and expected deaths for the same periods based on observations in 2018-19, accounting for projected changes in population size. Excess mortality ratios and expected mortality in the pandemic period are computed via Poisson regression models fitted to pre-pandemic (2018-19) and pandemic period (2020-21) observations, accounting for expected changes in population sizes (**Table S17**) via log offset terms. Bold text indicates totals across rows.

Table S16: Association of the proportion of excess deaths attributed to COVID-19 with ward-level deprivation index.

**Measure of COVID-19 associated mortality**	**β (95% UI)**			
	Early lockdown	*Wave 1*	*Wave 2*	*Total period*
Deaths among confirmed COVID-19 cases, as a proportion of all excess deaths	– –	–0.12 (–2.92, 2.23)	0.01 (–0.60, 0.71)	0.13 (–2.06, 2.30)
Deaths attributed to COVID-19, as a proportion of all excess deaths	– –	–0.19 (–5.10, 4.11)	0.02 (–1.22, 1.42)	0.32 (–5.37, 5.61)

UI: Uncertainty interval.
Excess deaths are estimated via the difference between observed deaths during 2020-21 and expected deaths for the same periods based on observations in 2018-19, accounting for projected changes in population size (**Table S17**).

Table S17: Data elements of the medical certificate of cause of death for hospitalized patients (Form 4) and non-institutional deaths (Form 4A).

Element	Sub-items
Name of deceased	
Sex	
Age at death	
	In completed years
	In completed months (if <1 year of age)
	In days (if <1 month)
	In hours (if <1 day)
Cause of death	
	Interval between onset and death, approximate
	Immediate cause—disease, injury, or complication which caused death, not the mode of dying such as heart failure, asthenia, etc.
	Antecedent cause—morbid conditions, if any, giving rise to the above cause, stating underlying condition last
	Other significant conditions contributing to the death but not related to the disease or conditions causing
	If the deceased was a female, was the death associated with pregnancy?
	If yes, was there a delivery?
Name and signature of the medical attendant certifying the cause of death	
Date of certificate	
Address of medical practitioner/medical attendant with registration number	

Table S18: Data elements of the death registration form.

Element
Name of the deceased
Sex
Date of birth
Name of spouse or parent
Address of death
Date of death
Residential address on the date of death
Ward number

Table S19: Expected annual rates of change in age- and sex-specific population sizes.

Age group	Fold change	
	Males	Females
0-9 years	0.9925	0.9938
10-19 years	0.9945	0.9948
20-29 years	1.0068	1.0041
30-39 years	1.0072	1.0049
40-49 years	1.0125	1.0108
50-59 years	1.0266	1.0274
60-69 years	1.0289	1.0310
70-79 years	1.0310	1.0318
≥80 years	1.0445	1.0473
**Overall**	**1.0089**	**1.0091**

Annual fold in population sizes by age- and sex-specific strata are obtained for Madurai District from the US Census Bureau (available from: https://www.census.gov/geographies/mapping-files/time-series/demo/international-programs/subnationalpopulation.html; accessed 22 March, 2023). Bold text indicates totals across rows.

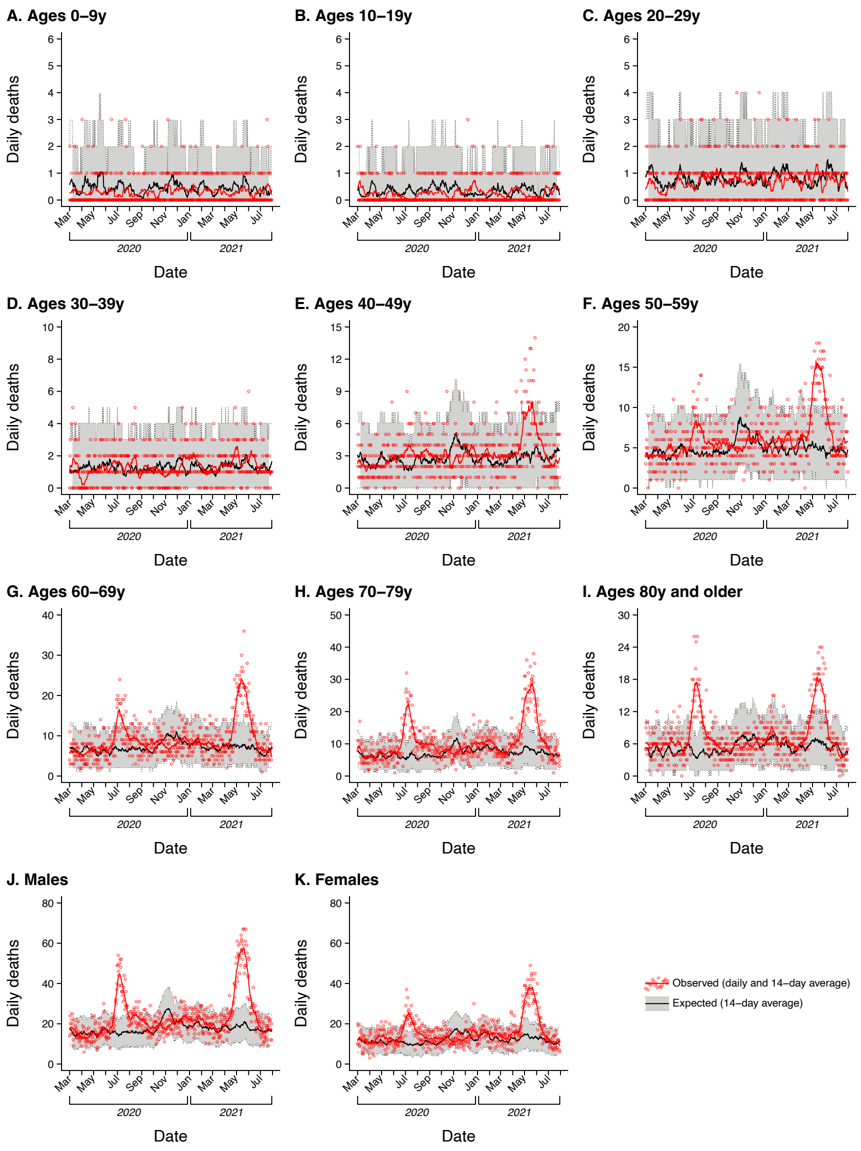

Figure S1: Observed and expected mortality across age- and sex-specific strata. We plot observed and expected deaths during the analysis period of March, 2020 to July, 2021, across age-specific (**a**-**i**) and sex-specific strata (**j**, **k**) We present observed deaths as red points with accompanying red lines indicating 14-day moving average values. Expected deaths (sampled via Poisson distributions fitted with 2 week moving-average mortality rates from 2018-19, accounting for changes in population size [**Table S17**]) are presented as black lines (median estimates) along with 95% uncertainty intervals (grey shading).

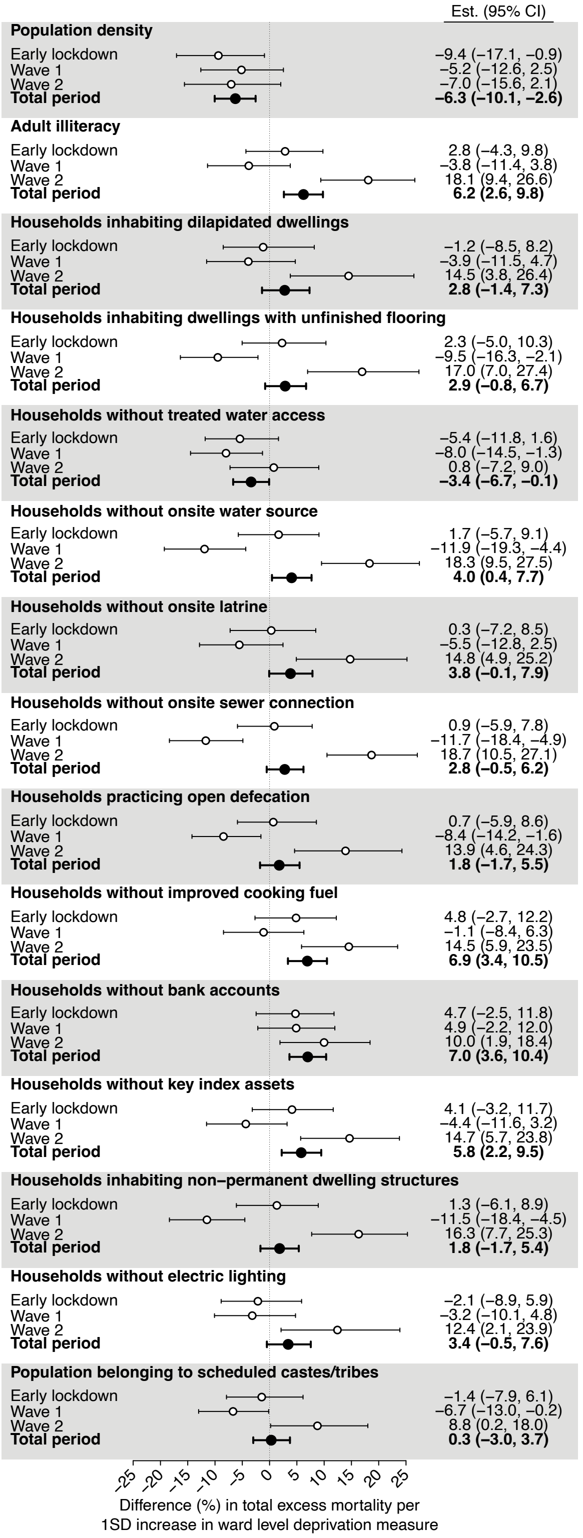

Figure S2: Association of excess deaths with specific ward-level deprivation indicators. We illustrate estimates of the association between excess deaths and a ward-level measure of community deprivation, as measured in the 2011 Census of India and used to construct the principal component measuring community socioeconomic deprivation for primary analyses (**Figure 3**). Values correspond to the absolute difference (in percentage-point units) in excess mortality, measured relative to expected deaths, associated with an increase by one standard deviation in the principal component-based measure of community deprivation. Lines denote 95% uncertainty intervals surrounding point estimates (medians), as estimated across regression models fitted across 10,000 independent draws from the distribution of the excess mortality outcome variable
