# Supplementary material for: Attributed causes of excess mortality during the COVID-19 pandemic in a south Indian city
Source: Nat Commun. 2023 Jun 15;14:3563. doi: 10.1038/s41467-023-39322-7 (PMC10272147; doi:10.1038/s41467-023-39322-7)
Supplement: Supplementary file 3 — Reporting Summary [file 41467_2023_39322_MOESM3_ESM.pdf]

nature portfolio

Corresponding author(s): Joseph Lewnard

Last updated by author(s): Jun 5, 2023

Reporting Summary

Nature Portfolio wishes to improve the reproducibility of the work that we publish. This form provides structure for consistency and transparency in reporting. For further information on Nature Portfolio policies, see our Editorial Policies and the Editorial Policy Checklist.

Statistics

For all statistical analyses, confirm that the following items are present in the figure legend, table legend, main text, or Methods section.

n/a	Confirmed	
☐	☒	The exact sample size (*n*) for each experimental group/condition, given as a discrete number and unit of measurement
☐	☒	A statement on whether measurements were taken from distinct samples or whether the same sample was measured repeatedly
☐	☒	The statistical test(s) used AND whether they are one- or two-sided<br>*Only common tests should be described solely by name; describe more complex techniques in the Methods section.*
☒	☐	A description of all covariates tested
☒	☐	A description of any assumptions or corrections, such as tests of normality and adjustment for multiple comparisons
☐	☒	A full description of the statistical parameters including central tendency (e.g. means) or other basic estimates (e.g. regression coefficient) AND variation (e.g. standard deviation) or associated estimates of uncertainty (e.g. confidence intervals)
☐	☒	For null hypothesis testing, the test statistic (e.g. *F*, *t*, *r*) with confidence intervals, effect sizes, degrees of freedom and *P* value noted<br>*Give P values as exact values whenever suitable.*
☒	☐	For Bayesian analysis, information on the choice of priors and Markov chain Monte Carlo settings
☒	☐	For hierarchical and complex designs, identification of the appropriate level for tests and full reporting of outcomes
☐	☒	Estimates of effect sizes (e.g. Cohen's *d*, Pearson's *r*), indicating how they were calculated

Our web collection on statistics for biologists contains articles on many of the points above.

Software and code

Policy information about availability of computer code

Data collection: No custom code or software was used for data collection and generation.

Data analysis: Analysis code is available from GitHub (https://github.com/joelewnard/Madurai_Deaths).

For manuscripts utilizing custom algorithms or software that are central to the research but not yet described in published literature, software must be made available to editors and reviewers. We strongly encourage code deposition in a community repository (e.g. GitHub). See the Nature Portfolio guidelines for submitting code & software for further information.

Data

Policy information about availability of data

All manuscripts must include a data availability statement. This statement should provide the following information, where applicable:

- Accession codes, unique identifiers, or web links for publicly available datasets
- A description of any restrictions on data availability
- For clinical datasets or third party data, please ensure that the statement adheres to our policy

Deidentified individual-level mortality data reported in this study are available via GitHub (https://github.com/joelewnard/Madurai_Deaths).

Human research participants

Policy information about studies involving human research participants and Sex and Gender in Research.

Reporting on sex and gender	Sex was recorded for all decedents. We present sex-stratified results in tables S3 and S4.
Population characteristics	Age and sex distributions of decedents are presented in tables S3 and S4; in total, 66.6% of deaths occurred in the community without healthcare supervision (Table S2). Causes of death are summarized in Table 2, and stratified for individuals with and without documentation of SARS-CoV-2 infection in Table S13.
Recruitment	Data for this analysis comprise all deaths notified to the Madurai Municipal Corporation under routine procedures for civil registration of births and deaths. No direct participant recruitment was involved.
Ethics oversight	This secondary analysis of data gathered through routine public health surveillance and civil registration procedures was considered exempt, non-human subjects research by the UC Berkeley Committee for the Protection of Human Subjects and the Princeton University Human Research Protection Program Institutional Review Board under NIH exemption category 4.

Note that full information on the approval of the study protocol must also be provided in the manuscript.

Field-specific reporting

Please select the one below that is the best fit for your research. If you are not sure, read the appropriate sections before making your selection.

☐ Life sciences ☒ Behavioural & social sciences ☐ Ecological, evolutionary & environmental sciences

For a reference copy of the document with all sections, see nature.com/documents/nr-reporting-summary-flat.pdf

Behavioural & social sciences study design

All studies must disclose on these points even when the disclosure is negative.

Study description	We analyzed all registered deaths in Madurai Corporation, India including demographic characteristics and attributed causes of death. We compared observed mortality (overall, by age- and sex-specific strata, and by attributed causes) to expectations under a continuation of 2018-19 patterns. All data used in this study are quantitative in nature.
Research sample	All deaths notified to the Madurai Municipal Corporation from March, 2018 through July, 2021. Of these deaths, 59.6% occurred among males; the proportion occurring at ages 0-9, 10-19, 20-29, 30-39, 40-49, 50-59, 60-69, 70-79, and 80+ years was 0.8%, 0.7%, 1.8%, 3.5%, 8.2%, 12.8%, 15.9%, 23.5%, 26.8%, and 18.9%, repectively. The city of Madurai was used for this analysis due to continuity of well-functioning vital surveillance in this setting throughout the pandemic. As the sample is universal (comprising all notified deaths), the data are representative of the distribution of deaths occurring in this jurisdiction, but may not represent the distribution of deaths in other cities or states within India, or in rural areas of India.
Sampling strategy	No sampling strategy was used. Civil registration systems in Tamil Nadu have previously been found to provide 100% coverage of deaths occurring within the state when compared against data collected through India's Sample-Based Registration System (validation described further in Rao & Gupta, The civil registration system is a potentially viable data source for reliable subnational mortality measurement in India; BMJ Global Health 5, e002586).
Data collection	Data for this study were generated through routine surveillance and reporting for cause-specific mortality via the Civil Registration System in Madurai. Vital surveillance in India is mandated under the Registration of Births and Deaths Act of 1969, which provides standardized elements for reporting of births and deaths. Operating procedures of Civil Registration Systems across India are highly decentralized, with the expectation that local registration units (states and districts) will develop customized strategies adapted for their unique contexts. In Tamil Nadu, a local Coordinating Committee leads vital surveillance within each district. Heads of affected households, in coordination with executive officers of lower administrative units (e.g., taluks, blocks, wards, or villages) have legal responsibility for notification of all births and deaths occurring in the community to their local registration unit. Police, community healthcare workers, operators of crematories or cemeteries, and others who may come into contact with the deceased have responsibility to report deaths that they are the first to observe.<br><br>Attending physicians have responsibility for reporting deaths occurring in healthcare facilities under their supervision, and for assigning medically-certified causes of death to these patients. In addition, physicians who provide care to individuals who die in the community have responsibility for assigning causes of death; causes of death for individuals who die in the community without prior care are assigned by registered medical providers at the point of declaring each death.72 Data included unique records for each death within Madurai Corporation during the periods of interest (2018-19 and 2020-21) abstracted from standardized MCCD forms (File S1, File S2) and CRS forms (File S3) including, for each decedent, their age, sex, date of death, ward of residence, attributed immediate cause of death, and name of the facility where the death occurred (for deaths occurring in healthcare facilities). These deaths data were recorded on written death certificates and an accompanying electronic database.<br><br>We used data from the 2011 Census of India to characterize socioeconomic attributes of wards within Madurai; whereas the census

is ordinarily carried out on a decennial basis, data collection for the 2021 Census of India was delayed to 2023 due to disruptions from the COVID-19 pandemic.

Researchers were not blinded to the study hypothesis or to data elements for this analysis.

Timing	Data cover all deaths notified from March, 2018 through July, 2021.
Data exclusions	No deaths were excluded from analysis.
Non-participation	As the study did not involve prospective recruitment, there was no circumstance under which an individual would decline participation.
Randomization	No interventions were administered, so no randomization was undertaken.

Reporting for specific materials, systems and methods

We require information from authors about some types of materials, experimental systems and methods used in many studies. Here, indicate whether each material, system or method listed is relevant to your study. If you are not sure if a list item applies to your research, read the appropriate section before selecting a response.

Materials & experimental systems

n/a	Involved in the study	
☒	☐	Antibodies
☒	☐	Eukaryotic cell lines
☒	☐	Palaeontology and archaeology
☒	☐	Animals and other organisms
☒	☐	Clinical data
☒	☐	Dual use research of concern

Methods

n/a	Involved in the study	
☒	☐	ChIP-seq
☒	☐	Flow cytometry
☒	☐	MRI-based neuroimaging
